# Association of Thoroughbred Racehorse Workloads and Rest Practices with Trainer Success

**DOI:** 10.3390/ani11113130

**Published:** 2021-11-01

**Authors:** Ashleigh V. Morrice-West, Peta L. Hitchens, Elizabeth A. Walmsley, Adelene S. M. Wong, R. Chris Whitton

**Affiliations:** Equine Centre, Faculty of Veterinary and Agricultural Sciences, Melbourne Veterinary School, University of Melbourne, 250 Princes Hwy Werribee, Melbourne, VIC 3030, Australia

**Keywords:** earnings, prizemoney, trainer, training, winnings, workload

## Abstract

**Simple Summary:**

Training workloads influence the risk of injury for racehorses, but veterinary advice to trainers is unlikely to be followed if it is associated with reduced racehorse performance, and thus their profitability. We therefore investigated whether the intended training programs for healthy horses was associated with the rate of wins, places and prizemoney earnt per start for Thoroughbred trainers in Victoria, Australia. Intended training workload was not associated with trainer prizemoney when other factors were taken into account, but more frequent rest breaks were associated with greater prizemoney per start earnt in the previous season. Intended trainer programs with moderate galloping distances as horses gain fitness for racing (i.e., not too high or too low compared to their peers), and moderate time between race starts were associated with better rates of wins and places. Workload associations with injury risk warrant further investigation, but these finding suggest that there is considerable scope for future modification of training workloads without negatively impacting trainer success rates and prizemoney earnings.

**Abstract:**

Understanding the relationship between the training practices of Thoroughbred racehorses and race performance is important to ensure advice given to trainers for injury prevention or management is practical and consistent. We assessed associations between intended volume and speed of gallop training (i.e., typical workloads for horses free of injury or other performance limiting conditions) and rest practices on official trainer career and previous season success rates (rate of wins and places, prizemoney per start). Sixty-six Australian Thoroughbred trainers were surveyed. Multivariable negative binomial regression models were employed for the outcomes career and previous season wins and places, and linear regression models for prizemoney per start. Intended training workload was not associated with prizemoney. Pre-trial total galloping distances (≥13.3 m/s) between 7500 m and 15,000 m were associated with a higher rate of career wins, and previous season wins and places per start (*p* < 0.05). Slow-speed (13.3–14.3 m/s) galloping distance to trial between 5000 m to 12,500 m was associated with higher rate of career placings per start, with reduced performance over 12,500 m (*p* = 0.003). Greater time between race starts was associated with a greater rate of previous season wins and prizemoney per start until three weeks between starts, with decline in performance thereafter (*p* < 0.05). Greater frequency of rest breaks was associated with greater prizemoney per start earnt in the previous season (*p* ≤ 0.01). These results suggest that modifications to training programs aimed at injury prevention, such as avoiding long galloping distances, should not adversely affect trainer success.

## 1. Introduction

A combination of cardiorespiratory fitness and musculoskeletal adaptation is required to enable career success and longevity of racehorses. The volume and type of workload that optimises racing performance is unknown. This is reflected in the lack of universality in training regimens implemented by Thoroughbred trainers [1]. Both high and low workloads are associated with musculoskeletal injury (MSI) in Thoroughbred racehorses, demonstrated predominately in investigations of distal and proximal limb fatal and non-fatal fractures [2,3,4,5,6,7,8,9,10], but also in studies of superficial digital flexor tendon injuries [11], suspensory apparatus failure [11], and dorsal metacarpal disease [12]. These relationships indicate that workload modification may contribute to MSI rate reduction. However optimising workloads to prevent injury is unlikely to have a high uptake by trainers if these strategies compromise performance outcomes.

A correlation between longer distances exercised at sub-maximal speeds and greater race winnings has been observed [13,14,15]. A quadratic relationship between distance worked in training and racing and performance has been reported, with both higher and lower gallop distances associated with poorer performance outcomes [14]. But when examining the effect of training volume on performance in the individual horse, the confounding effect of injury on workload needs to be considered. This “healthy horse effect” has been described whereby horses with less underlying injury undertake higher workloads [10].

Along with the workloads of race-fit horses, other training practices may affect horse performance. These include the effect of the rapidity of progression from unfit to race-fit, or of rest frequency and duration, which have not been investigated previously.

The present study aims to identify associations between intended volume and speed of gallop training (i.e., the typical workload a trainer would use for a horse free of injury or other performance limiting conditions), and duration and frequency of rest periods reported by Victorian Thoroughbred trainers on horse performance and thus trainer success. We hypothesise that there is an optimal workload volume range whereby (1) too high or too low maintenance gallop workloads, (2) too rapid progression or too high workloads in the lead up to a barrier trial (a practice race supervised by racing stewards on an official racetrack, which is typically undertaken at least once at the start of a horse’s career and at the start of each race campaign after a rest period), and (3) too short or low frequency of rest periods are associated with poorer performance.

## 2. Materials and Methods

### 2.1. Population, Sample and Study Design

Sixty-six (of 889 registered) trainers in Victoria, Australia representative of all licence levels (Class of trainers categorised as: Class A, General, Restricted) and regional classifications (Metropolitan, Provincial, Country) participated in a survey (2016/2017 race season). The full survey methods of trainer-reported Thoroughbred racehorse training regimens have been described previously [1], and are summarised in Appendix A.

### 2.2. Predictor Variables

Trainer data included license level (trainer class), regional classification and the number of horses in training at the time of survey. Information on ages and general intended race distance (sprint, middle distance, long distance; “staying”) of horses trained by each trainer were recorded. Using the survey information, a dataset was generated for each trainer that included the typical training track surface, speed (m/s) and distance (m) for each age and intended race distance of their horses both pre-trial (progressive workloads from paddock fitness) and maintenance workloads (workloads for maintaining fitness between races during a racing campaign; m/month), and typical frequency and duration of rest periods for horses under their care (in the absence of injury or illness), for the different categories of horses for each trainer, Appendix A. Additional pre-trial variables included number of weeks from resting to trial, number of weeks in slow versus fast workouts, and designated program type based on cluster analyses previously undertaken: (1) high volume with large amounts of slow-speed gallop; (2) moderate volume and (3) fast and light programs-low workloads over the shortest time period [1]. Maintenance workload programs were categorised based on cluster analysis as (1) low volume; (2) medium volume; (3) medium volume with greater high-speed work; and (4) high volume [1]. Trainers were ranked according to their maintenance workload programs and distribution of horses in their stable, detailed methods are in Supplementary Methods S1. We defined galloping workloads as distances (m) at speeds of 13.3 m/s and above (“total galloping”), and categorised them into four speeds (13.3–14.3 m/s “slow-speed galloping”, 14.4–15.4 m/s “medium-speed galloping”, 15.5–16.7 m/s “high-speed galloping”, ≥16.8 m/s “very high-speed galloping”).

### 2.3. Outcome Variables (Measures of Trainer Success)

Number of wins, places, and prizemoney were key trainer-level measures of success identified by a systematic-review of racehorse performance [16]. Total career and previous season (1 August 2016 to 31 July 2017) race-data for each of the 66 trainers (wins, places, prizemoney in Australian dollars [AUD], number of race starts, race distance, race class) were obtained through the official repository for Australian racing results. (See: Racing Victoria 2017, www.racing.com (accessed on 13 to 29 March 2018)). Previous season results were assessed in addition to career success rates to investigate potential evolution of training methodologies over time, particularly in the case of trainers with decades of experience. Results for race starts by specific race distances (≤1200 m, 1201–1400 m, 1401–1600 m, 1601–2000 m, >2000 m) and elite race grades (Group 1–3, Listed) were obtained. Distances were further categorised into sprint (≤1400 m), middle distance (1401–2000 m), and staying races (>2000 m).

### 2.4. Data Analysis

Data analyses were conducted using Stata/SE version 15.0. (StataCorp. 2017. Stata Statistical Software: Release 15. College Station, TX, USA: StataCorp LP.) Continuous variables were assessed for normality using histograms and Shapiro-Wilk tests. Descriptive statistics are reported as means and standard deviations (SD) or medians and interquartile ranges (IQR) as appropriate.

Multiple observations on the same trainer were condensed into summary data on training methods with adjustment using robust standard errors to account for clustering at the trainer-level. Univariable analyses were performed for the six outcomes: trainer career wins, career places, career prizemoney per start, previous season wins, previous season places, and previous season prizemoney per start (Models 1–6, respectively). Regression models for the predictor variables on each of the six trainer success outcome measures were generated. Models for trainer career and previous season wins and places (Models 1, 2, 4, 5) were generated using Negative Binomial regression given the overdispersion generated when a Poisson model was employed (where overdispersion implies greater variation than predicted by the model, i.e., variance larger than the mean) [17]. Improvement in model fit as a Negative Binomial model compared to the alternative simple Poisson was confirmed using Likelihood Ratio Tests, deviance and Pearson’s chi-squared statistics. We modelled the number of wins or places (outcome) and offset this with the logarithm of the number of starts (exposure), effectively giving a rate of wins or places per start by trainer (i.e., number of wins or places divided by the number of horse starts) [18]. Models for career and previous season prizemoney per start (Models 3, 6) were conducted using linear regression. These models utilised a transformed outcome variable of the natural log of prizemoney per start to improve residual normality, and this practice has been similarly adopted in previous race performance data analytics [14]. For trainers where previous season prizemoney was zero, 1 AUD was substituted. Linear regression model residuals were checked for normality via Shapiro-Wilk tests and assessing histograms. Natural log (number of horses) and quadratic (total gallop distance, distance at 13.3–14.3 & 15.5–16.7 m/s trained prior to trialling, frequency of racing) transformations were applied to continuous predictor variables based on a departure from linearity when investigating their association with outcome variables.

Spearman correlations (*r*) were used to assess the degree of collinearity between predictor variables. Trainer categorical variables (license, region, cluster groups) were considered as ordinal variables for the purposes of correlation calculations.

Variables with *p* ≤ 0.2 in univariable screening were considered in multivariable models and retained where *p* ≤ 0.05 using backwards and forward stepwise elimination. Where there was a strong correlation between univariably significant predictor variables (*r* > 0.6) only one correlated predictor at a time was included in the potential models to avoid multi-collinearity. Model fit was assessed by minimisation of AIC and BIC values. Biologically plausible first-order interaction terms were screened for statistical significance and assessed graphically. For linear regression models, coefficients and their 95% confidence intervals (95% CI) are reported. For Negative Binomial models, coefficients are reported in their exponentiated form (Incident Rate Ratios, IRR).

As monthly reported distances galloped (maintenance workloads) were not uniform across each training stable and depended on the intended race distance and class of horse, sub-set univariable analyses were performed. Subsets were horse program type (sprint, middle, stayer, elite) based on the previous season performance results for the grouped race distances and elite race grades. The intention of running the subset analyses was to assess for associations between various workload factors and trainer success that might vary according to different race distances or be specific to elite race. Results are presented adjusted for stable size.

## 3. Results

### 3.1. Descriptive Statistics

Licenced trainers had a median 1220 career race starts (IQR 261–3409), and 108 previous season race starts (IQR 33–244). Median career prizemoney was 3500,000 AUD (IQR 450,000–13,000,000) and last racing season prizemoney was 1300,000 AUD (IQR 630,000–2,200,000). Trainers had a median of 140 career wins (IQR 22–40), 380 career places (IQR 70–1218), 10 previous season wins (IQR 4–31) and 36 previous season places (IQR 7–85). Trainers had a mean rate of 0.11 (SD 0.04) career wins, 0.31 (SD 0.08) places, and 0.10 (SD 0.05) previous season wins and 0.31 (SD 0.12) places per start.

### 3.2. Univariable Results

Univariable associations between predictor variables and trainer success outcomes are presented in Supplementary Materials Table S1 for career wins (Model 1), places (Model 2), prizemoney (Model 3), previous season wins (Model 4), places (Model 5), and prizemoney (Model 6). Univariably significant (*p* < 0.05) workload to trainer success associations are shown in Supplementary Materials Figure S1. Maintenance cluster group and ranked maintenance training intensity were not associated with trainer success in any models (Supplementary Materials Table S1).

Weak to moderate correlations between trainer-category predictor variables were present: experience of trainers increased with larger stables (*r* = 0.62) and from country to provincial to metropolitan areas (*r* = 0.42; *p* < 0.01). Trainers with more horses tended to have higher rates of weekly distance accumulation and higher total monthly distances for maintenance programs (*r* = 0.29; *p* = 0.02). Pre-trial total gallop and the slow-speed gallop (13.3–14.3 m/s) distance variables were strongly correlated (*r* = 0.90, *p* < 0.001). Increasing pre-trial gallop distance correlated with maintenance workloads based on trainer ranking (from low to high) (*r* = 0.68, *p* < 0.001) and maintenance workload cluster group (*r* = 0.70, *p* < 0.001). Designation of trainers into pre-trial cluster groups was moderately correlated with maintenance cluster group classification (*r* = 0.45, *p* < 0.001).

### 3.3. Multivariable Results

**Figure 1:**
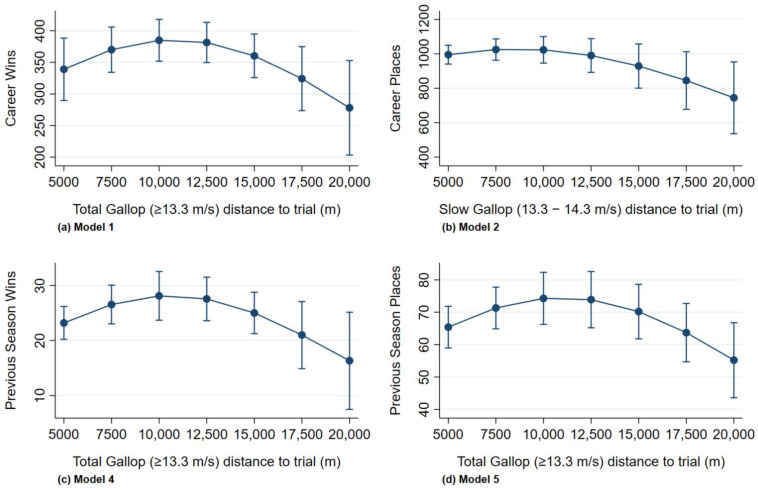
Quadratically transformed workload variables (as galloping distance in preparation for trialling) significant in multivariable negative binomial regression model’s for trainer success as (**a**) career (Model 1) wins and (**b**) (Model 2) places, and (**c**) previous season (Model 4) wins and (**d**) (Model 5) places from 66 surveyed Victorian Thoroughbred trainers, accounting for an exposure variable of number of starts.

Multivariable associations are presented in Table 1, with prediction plots for the quadratic transformations for galloping workloads displayed in Figure 1. Pre-trial total galloping distances (>13.3 m/s) between 7500 m and 15,000 m were associated with a greater rate of career wins (Model 1 *p* = 0.02) and previous season wins and places (Models 4, 5 *p* < 0.05, Figure 1). Total galloping distance to trial was interchangeable in the rate of wins multivariable Model 1 with its co-linear variable slow-speed galloping distance to trial (13.3–14.3 m/s). Pre-trial slow-speed galloping distance of 5000 m to 12,500 m was associated with greater rate of career placing, with reduced career placings for distances greater than 12,500 m (Model 2 *p* = 0.003). For previous season wins and prizemoney, horse performance per start improved with increasing spacing of races until three weeks between starts, with a decline in performance thereafter (*p* < 0.05). Higher frequency of rest periods was associated with greater previous season prizemoney (*p* = 0.01). Larger stable sizes were associated with a greater rate of career wins and places and previous season prizemoney (*p* < 0.01), and larger stable size was the only variable associated with greater career prizemoney per start (Model 3 *p* < 0.001).

### 3.4. Subset Analysis for Success in Specific Race Types

In a univariable subset analysis, trainer success models for sprint training programs were not affected by maintenance gallop distances, and no group’s workloads (race distance categories or elite training programs) were significantly associated with prizemoney per start (Supplementary Materials Table S2). After adjusting for stable size the association for high-speed galloping distance did not retain significance for trainers’ middle-distance success rates, with stable size significantly associated with trainer success over this race distance. Models for staying horse programs, adjusted for stable size, showed a modest association of increased slow-speed galloping (13.3–14.3 m/s) with increased rate of places (IRR 1.02; 95% CI 1.00, 1.05; *p* = 0.02). However, there were small but opposite effects of high-speed galloping and very high-speed galloping; higher win rates were associated with both lower and higher volumes of high-speed galloping (IRR 1.01; 95% CI 1.01, 1.02; *p* < 0.001), whereas higher win rates were associated with mid-volumes of very high-speed galloping, with win rates reduced for lower and higher volumes (IRR 0.99; 95% CI 0.99, 1.00; *p* = 0.01). Although, the proportion of trainers exercising stayers at very high-speeds was small (*n* = 6).

For elite horse programs adjusted for stable size, places were increased with increased maintenance total gallop distance (>13.3 m/s, IRR 1.06; 95% CI 1.01, 1.10; *p* = 0.01; win rate *p* = 0.06), and wins increased as fast-galloping (15.5–16.7 m/s) increased, with a reduction for the highest distances of fast-galloping (IRR 0.94; 95% CI 0.90, 0.98; *p* = 0.01).

## 4. Discussion

We investigated associations between intended training practices and racing success for racehorse trainers in Victoria, Australia. There were few multivariable associations between training workloads and trainer success rates. Intended training workloads had no association with trainer prizemoney outcomes when other factors were taken into account, whereas the trainers who utilised the lowest and highest total or slow-speed galloping workloads as horses gained fitness and prepared for racing had fewer wins and places. Similarly, the lowest and highest racing frequency was associated with less success in last season, with 2.5 to 3 weeks between race starts appearing to be optimal.

Pre-trial galloping workloads were associated with trainer success, with horse performance compromised for those that galloped less or more than most of the surveyed cohort. In the pre-trial period slow-speed galloping correlated strongly with total distance galloped which likely reflects the typical Australian training pattern of a high proportion of gallop workouts at “even-time” (15 s/200 m; 13.3–14.3 m/s), with a short sprint finish (typically high-speed and above) [1]. This is consistent with total galloping and “even-time” galloping being the predominant distance measures associated with trainer success. The pre-trial short sprint distance (high or very high speeds to the winning post in training) comparatively held no multivariable associations. Galloping exercise is necessary for appropriate bone adaptation to tolerate racing loads, but our findings suggest there is no benefit to racing success through extended high-speed galloping pre-trial [19,20,21]. Further work is required to elucidate the optimal ranges for introduction of high-speed galloping relative to performance and injury.

We found few associations between trainers’ intended maintenance workloads and racing success, with no associations based on stable-level data. Previous research examining actual distance galloped by horses in training in the UK found that greater high-speed distances of combined training and race gallops in the previous 30 days was associated with a greater likelihood of winning a race or earning prizemoney [14]. For horses that earnt prizemoney in that study, a quadratic association of high-speed galloping volume was found whereby horses galloping less than ~4800 m/month earnt more prizemoney for increasing canter distances, but horses galloping > 4800 m/month had a decline and then plateau in earnings with greater slow-speed exercise [14]. Although similar to our pre-trial workload findings which were to some extent correlated with maintenance workloads, maintenance factors which related to a trainer’s overall training intensity had no association with racing success. The UK study was likely to be influenced by a healthy-horse effect with horses that were performing well and remaining injury free training and racing more, whereas we report findings on intended rather than actual workloads in an effort to minimise the direct effect of injury. The discrepancy may also be a result of differences in training and racing environments and/or training philosophies and methodologies in different racing jurisdictions. Additionally the UK study reported a quadratic effect of distance raced in the previous 30 days on prizemoney with a reduction over cumulative race distances of ~10,000 m, and horses were more likely to win a race if they had raced in the preceding 30 days [14]. We considered the frequency of racing which could be regarded as a proxy for racing intensity at the stable-level, where moderate time between races was associated with improved success rates. This relationship was univariably (but not multivariably) found in the UK study, with two starts in a 30 day period associated with greater prize money compared to one start, but horses that raced four or more times earnt less prizemoney per start [14]. Other studies examining intensity of racing campaigns on performance have assessed the effect of individual horses’ frequency of racing on career duration, but have produced differing results. Increased time between starts was associated with longer careers according to official race records [22], however in horses’ 2-year-old year, more frequent racing and for females less frequent racing have both been associated with increased career duration [23,24].

It is possible that the effect of workload on performance differs for horses prepared for different race lengths. For instance, there are varying energy and oxygen consumptive requirements for horses racing over different distances [25]. We therefore conducted a subset analysis to stratify trainer success by intended programs for horses targeted to specific races. We previously demonstrated that in this cohort, sprinters were trained over shorter distances at slow-speed gallops but there was no difference in high-speed galloping by intended race distance [1]. In the current study we found no association between intended maintenance galloping workloads and trainer success measures for sprint horse programs. After adjusting for stable size, places for race starts in staying races were mildly improved by greater maintenance distances of slow-speed galloping. Both the longest and the shortest gallop distances at high-speed (15.6–16.7 m/s) for staying horse programs were associated with better trainer success than mid-distance workloads at high-speed. There may therefore be little benefit in extended workloads. The univariable association between greater slow-speed galloping distances and racing success for elite horse programs, after adjusting for stable size, may reflect the potential for elite horses to tolerate greater workloads, but we cannot rule out other reasons for this observation.

A greater number but not longer duration of rest periods was associated with greater prizemoney per start. There is little published on the effect of rest on horse performance. Periods without training have demonstrated benefits on rates of bone remodelling and therefore replacement of fatigued bone accumulated during active racing campaigns [26]. Our finding suggests that increasing the total amount of rest is best achieved by adding breaks from training rather than increasing the duration of each rest period. This relationship warrants further investigation.

Greater trainer stable size and univariably metropolitan stable locations were associated with improved trainer success rates. These findings corroborate results from Thoroughbred racing performance in New Zealand and that it was important to correct for stable size and location in the multivariable analyses [27,28]. Similarly race earnings in Poland were clustered at the trainer-level, with increasing stable size having the most substantial association with performance [24]. In both Australian and UK racing jurisdictions both horse and trainer have been associated with race performance, even after accounting for different workloads between trainers [15,29].

Our findings and those of others are consistent with the lowest and highest volumes of galloping not being conducive to maximising horse performance. However, in horses in this study it was the volume of galloping in preparation for training that demonstrated that association rather than gallop volumes once horses were fit to race. It is possible low workloads are not sufficiently preparing horses for race-level fitness and that horses trained over long distances are over-trained. Overtraining is a phenomenon described most extensively in human athletes and military recruits, but also in equine athletes mostly in Standardbred racehorses, whereby beyond a certain level of activity without sufficient rest an individual does not continue to adapt productively to training. The syndrome is characterised by decreasing performance capacity, often associated with elevated blood lactate, elevated heart rates during exercise, reduced appetite and/or weight loss, changes in behaviour and reluctance to undertake strenuous exercise [30,31,32,33,34]. Human studies also report muscle pain and fatigue as well as increased injury susceptibility [35]. In this Thoroughbred population, overtraining could indicate similar physical and behavioural changes, with potential underlying muscle, joint, and/or bone pain. Some trainer’s low workloads in preparation for trial may also be offset by using races to gain fitness which would present as poorer horse performance at the beginning of campaigns. Whilst pre-trial workloads were consistent within stables, maintenance workloads were highly variable for different types of training programs. At the stable-level, in multivariable analysis for intended maintenance workloads we could only assess ranking of training intensity and clustered workload levels. Had we assessed the maintenance workloads of individual horses, we may have been able to more closely mimic the findings of previous studies.

This study is subject to the limitations of surveys, where errors typically result either through either measurement/processing faults (observational errors) or inadequate sample selection where the study population is not a true representation of the total population (non-observational errors) [36,37]. We did not have information on all external factors (e.g., race conditions, jockey influence, trainer resources etc) which could potentially confound or influence the association between study factors and success measures.

As per the preceding companion paper, training and rest period data was obtained via in-person interviews and represents holistic training approaches to a range of horses in each stable rather than verified workload data from specific horses [1]. Injuries directly compromise workload in addition to horse performance. By looking at success rates across entire stables and assessing intended workloads, we aimed to reduce the effect of individual horses’ inability to cope with prescribed programs. The present study did not assess injury rates which clearly need careful consideration when assessing any perceived benefit to racing performance. The associations between intended workloads and trainer injury rates for this cohort is under investigation and will be presented in a companion paper.

The study is subject to potential misclassification bias and recall bias given the retrospective nature of the training data collected [38,39]. However, in-person interviewing enabled greater detail to be collected for a large number of questions compared to a larger online survey, and minimised nonresponse errors (where some participants may have otherwise only answered part of the questionnaire). All trainers were assured of the confidentiality of the study and that results would not be published or presented to the racing authority on an individual identifiable basis, therefore we are confident that the answers provided were truthful. Moreover, the limited literature on effects of workloads on horse injury or performance meant that trainers were unlikely to be biased by what they believed the “correct” answer to be. The study is also subject to potential selection bias as despite being advertised to the entire training population, the study was voluntary and participant selection was not entirely random which could therefore compromise the generalisability of findings to the wider state or nationwide population. Finally, the relatively small sample size (7.4% response rate) and the multi-collinearity of predictor variables, specifically with inter-changeability between workload variables and stable size, led to difficulty fitting multivariable models. Sample size calculations were prospectively conducted with the intention of having sufficient power to detect differences in workloads between groups of trainers [1]. The presented results are aggregated from individuals, but modest second-order effects were identified at the trainer-level. Given the clear inter-horse variation in racing performance variation, future investigations at the individual horse level may identify much greater effects.

## 5. Conclusions

When advising trainers on management practices that will minimise musculoskeletal injury, it is critical that the impact of that advice on success and performance is considered. Here we found limited data linking intended training programs or rest factors in the management of Thoroughbred racehorses with trainer success indicated by wins, places or earnings per start. Only the very low or very high intended workloads were associated with poorer trainer success rates, but not consistently. Therefore there may be substantial potential for manipulation of workload quantity and intensity without the risk of compromising trainer, industry personnel or horse owner earnings.

## Figures and Tables

**Table 1:** Multivariable modelling of career and previous season trainer racing success outcomes from 66 surveyed Victorian thoroughbred trainers. Wins and places were analysed as negative binomial models for the number of wins/places with an exposure variable of the number of starts and presented as exponentiated coefficients to show Incident Rate Ratios (IRR’s) and their associated 95% Confidence Intervals (CI). Prizemoney per start outcome variables were log-transformed and analysed via linear regression and therefore presented as point estimates and their associated 95% CI’s.

	Career			Previous Season		
	Model 1	Model 2	Model 3	Model 4	Model 5	Model 6
	Wins	Places	Prizemoney	Wins	Places	Prizemoney
	IRR (95% CI)	IRR (95% CI)	Coef. (95% CI)	IRR (95% CI)	IRR (95% CI)	Coef. (95% CI)
Stable size (number of horses)	1.15 (1.04, 1.28) **	1.12 (1.05, 1.19) ***	0.50 (0.40, 0.60) ***			2.72 (1.84, 3.59) ***
Gallop distance (>13.3 m/s) ^a^ to trial *x*	1.09 (1.01, 1.17) *			1.14 (1.10, 1.29) *	1.08 (1.02, 1.15) *	
*x* ^2^	0.996 (0.99, 0.999) *			0.99 (0.988, 1.00) *	0.996 (0.99, 0.999) **	
Slow-speed gallop distance (13.3–14.3 m/s) ^a^ to trial *x*		1.04 (1.01, 1.08) **				
*x* ^2^		0.998 (0.996, 0.999) **				
Weeks between starts *x*				3.35 (1.02, 11.06) *		10.50 (0.38, 20.62) *
*x* ^2^				0.80 (0.64, 0.99) *		−2.34 (−4.26, −0.43) *
Number of rest periods/year						2.38 (0.70, 4.05) **

* *p* < 0.05, ** *p* < 0.01, *** *p* < 0.001. ^a^ For a 1000 m change in cumulative distance.

**Figure A1:**
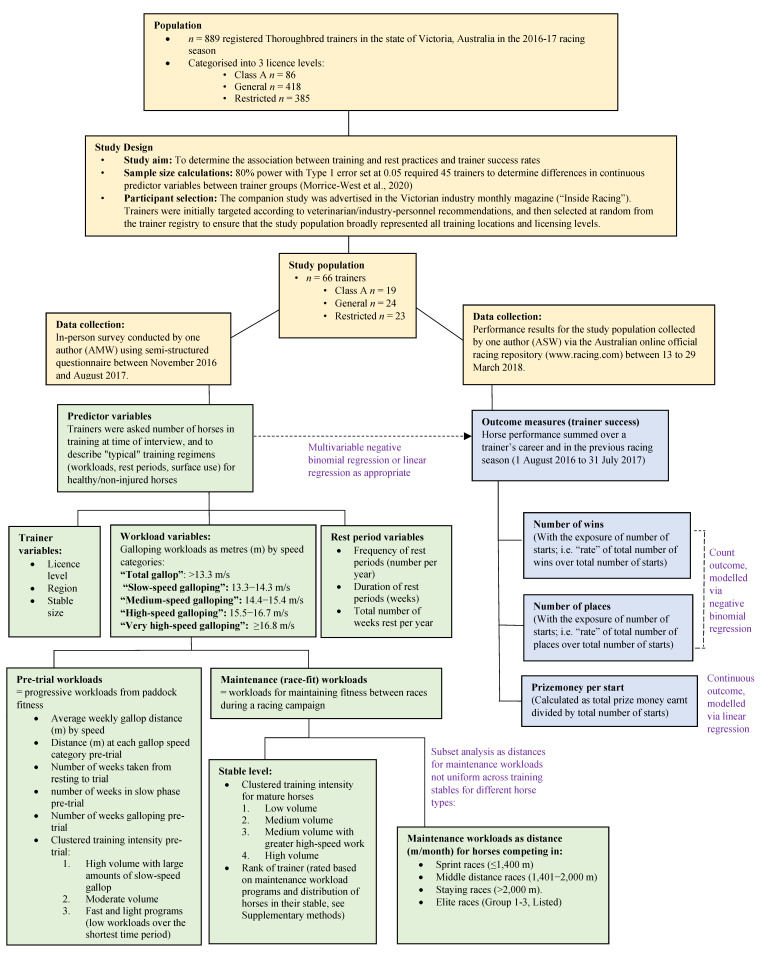
Study design for investigating the effect of high-speed training volume, and duration and frequency of rest periods on the Thoroughbred racing success rates, using a surveyed cohort of 66 Thoroughbred trainers in Victoria, Australia. Yellow boxes denote the overall study design and data sources, green boxes denote predictor variables, blue boxes denote outcome variables, and purple comments denote the relevant modelling methodology.

## Data Availability

The data presented in this study may be available on request from the corresponding author. The data are not publicly available due to privacy reasons related to potential for individual trainer identification.

## Supplementary Materials

The following are available online at https://www.mdpi.com/article/10.3390/ani11113130/s1, Methods S1: Supplementary methods, Table S1: Univariable regression models of career and previous season trainer success outcomes from 66 surveyed Victorian thoroughbred trainers. Wins and places were analysed as negative binomial models for the number of wins/places with an exposure variable of the number of starts and presented as exponentiated coefficients to show IRR’s and their associated 95% Confidence intervals (CI). Prizemoney per start outcome variables were log-transformed and analysed via linear regression and therefore presented as point estimates and their associated 95% CI’s., Figure S1: Quadratically transformed workload variables (as galloping distance (m) in preparation for trialling at various speeds, and frequency of racing as weeks between starts) significant in univariable models for trainer success as career wins (Model 1), places (Model 2) and prizemoney per start in Australian dollars (AUD) (Model 3), and previous season wins (Model 4), places (Model 5) and prizemoney per start in AUD (Model 6) from 66 surveyed Victorian Thoroughbred trainers. For Models 1, 2, 4 and 5 we generated as negative binomial regression models accounting for an exposure variable of number of starts. Prizemoney per start (Models 3 and 6) were analysed with log transformed outcome variables and back transformed for graphical interpretation purposes accounting for variance in the standard errors of the models. Model 6 graphs are presented excluding the trainers who did not earn prizemoney in the previous season, Table S2: Previous season trainer success outcomes as wins, places and prizemoney per start (log transformed) according to monthly distances galloped by subsets of horses within each training stable based on intended race distance from short to long distance (sprint/middle/stayer) and elite horses from a surveyed cohort of 66 Thoroughbred trainers in Victoria, Australia.

## Appendix A

Study design for investigating the effect of high-speed training volume, and duration and frequency of rest periods on the Thoroughbred racing success rates, using a surveyed cohort of 66 Thoroughbred trainers in Victoria, Australia.
