# Supplementary material for: Association of Thoroughbred Racehorse Workloads and Rest Practices with Trainer Success
Source: Animals (Basel). 2021 Nov 1;11(11):3130. doi: 10.3390/ani11113130 (PMC8614314; doi:10.3390/ani11113130)
Supplement: Supplementary file 1 [file animals-11-03130-s001.zip › Supplemetary methods_S1.pdf]

Supplementary Methods S1 - Trainer ranking for race-fit maintenance workloads

Each trainer was grouped into their primary workload level (the level in which the majority of their reported maintenance programs were categorised as). Where trainers had equal proportions of programs across two groups (n = 4) the group to which the volume of high-speed gallops most closely matched was selected. Trainers were ranked according to the proportion of maintenance workloads in each of the described cluster volume groups from lowest to highest training intensities. For each trainer, we generated a standardised score as follows. The proportion of the stable trained at each intensity level was multiplied by cluster groups' median total monthly gallop distance, a z-score was then generated and converted into a percentage. For example, a trainer with low level programs across all of the horse demographics in his stable would have resulted in 100% of programs at 4,800m/month, and thus being ranked in the 1st percentile of workload intensity compared to the cohort. Other maintenance workload variables included frequency of racing (number of weeks between starts). Rest period variables included frequency of rest periods per year, total number of weeks in rest per year, and a rank based on the total annual weeks in rest (as least number of weeks off per year to highest weeks off per year).
