# Supplementary figures and images for: Association of Thoroughbred Racehorse Workloads and Rest Practices with Trainer Success
Source: Animals (Basel). 2021 Nov 1;11(11):3130. doi: 10.3390/ani11113130 (PMC8614314; doi:10.3390/ani11113130)

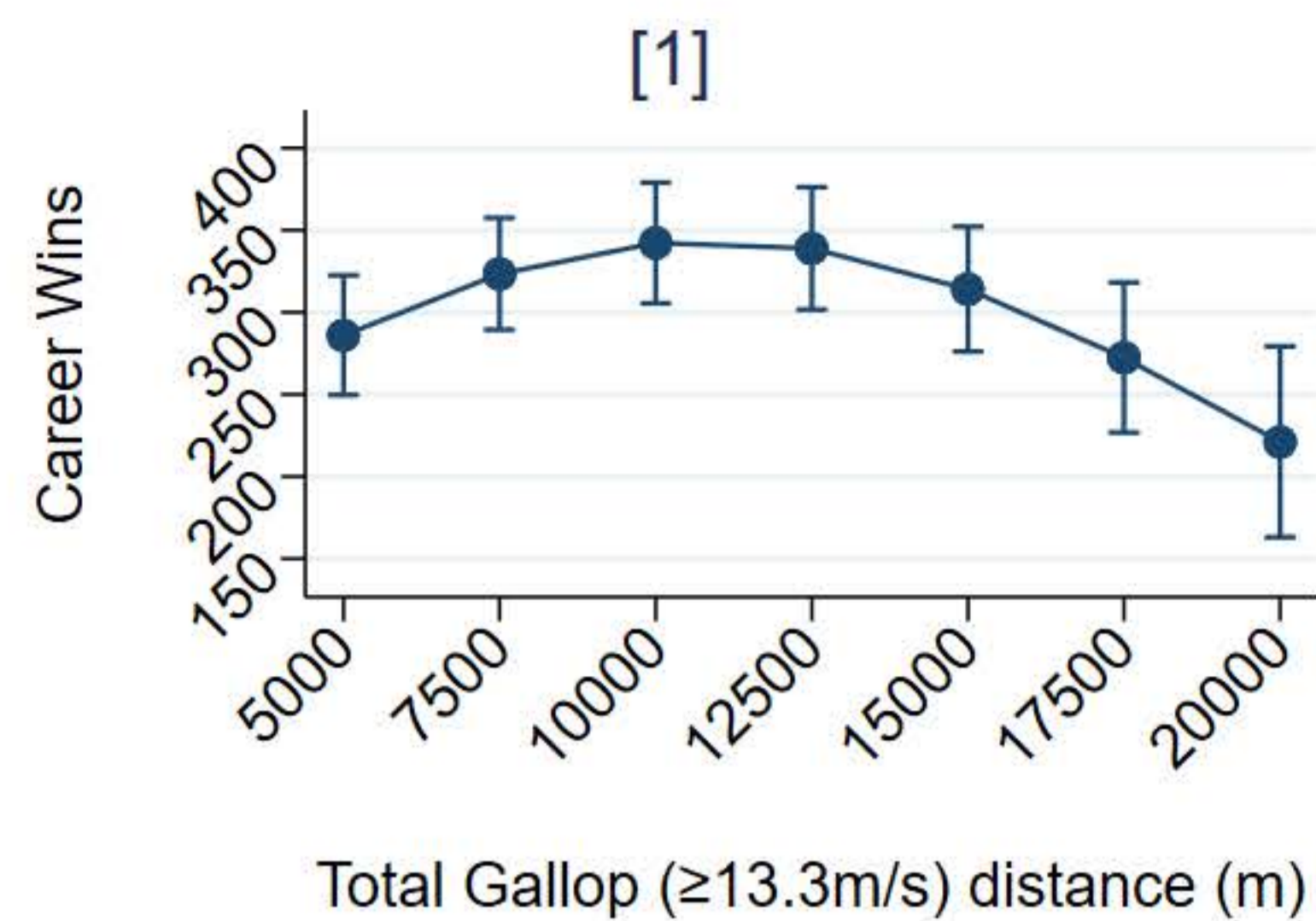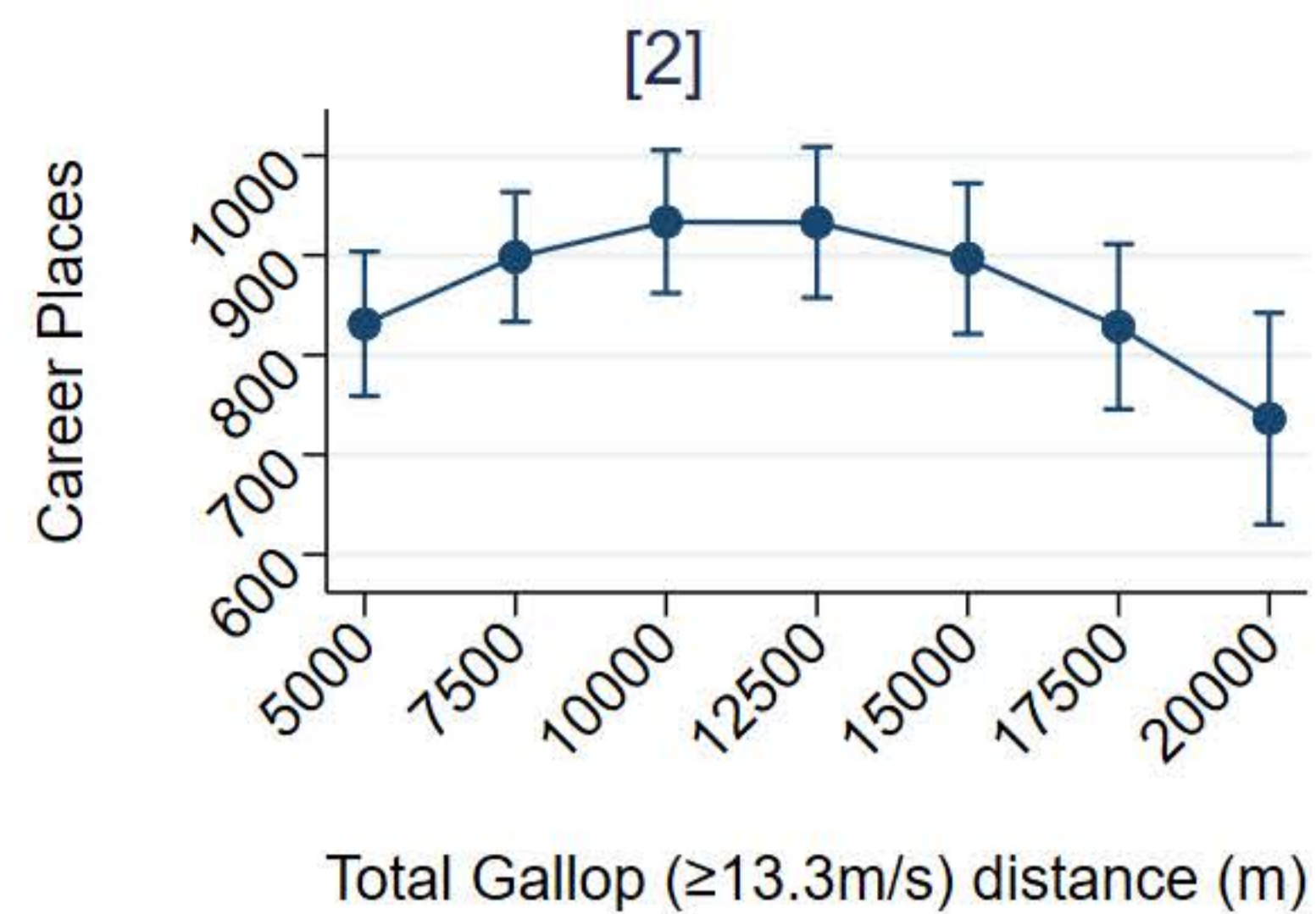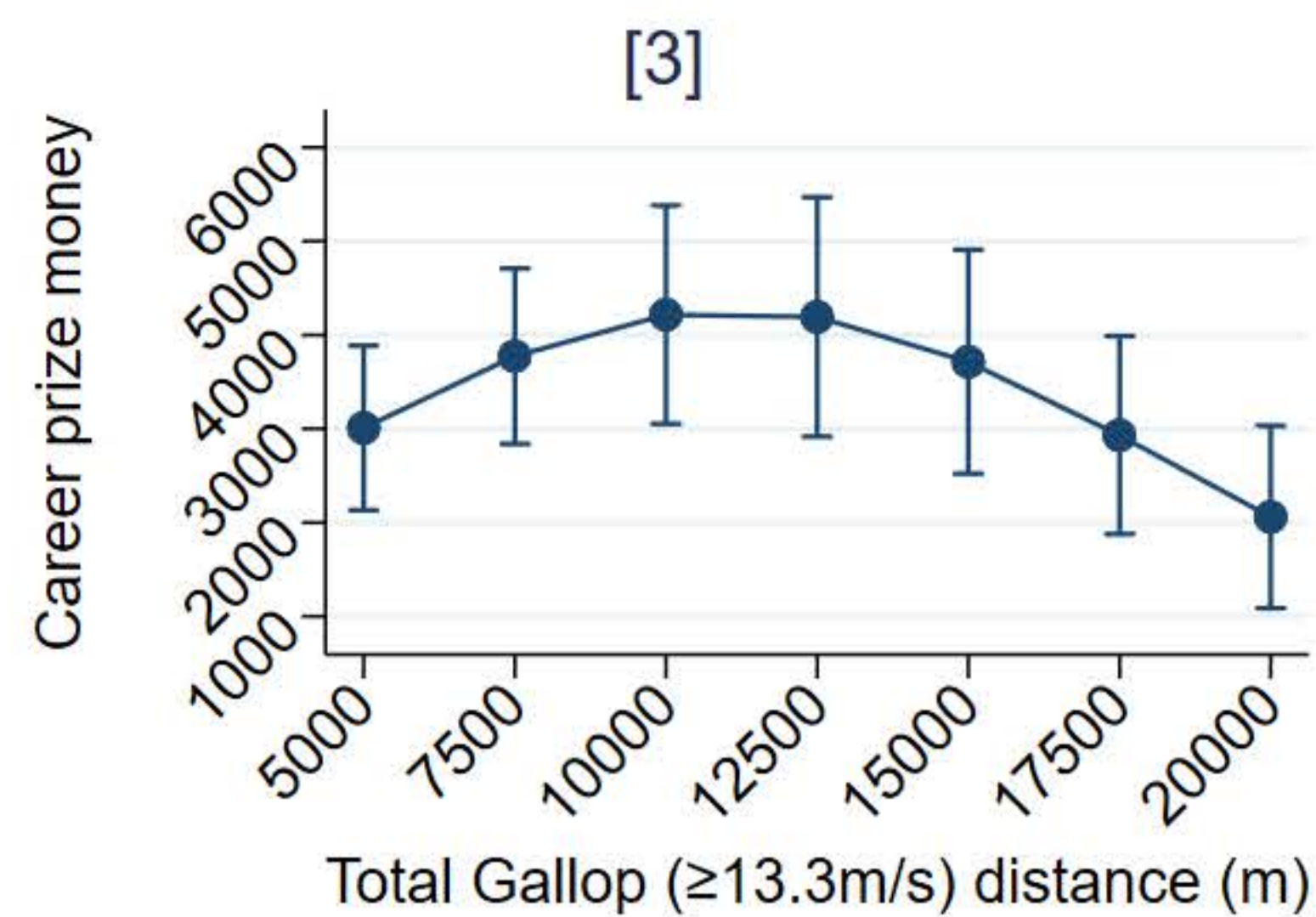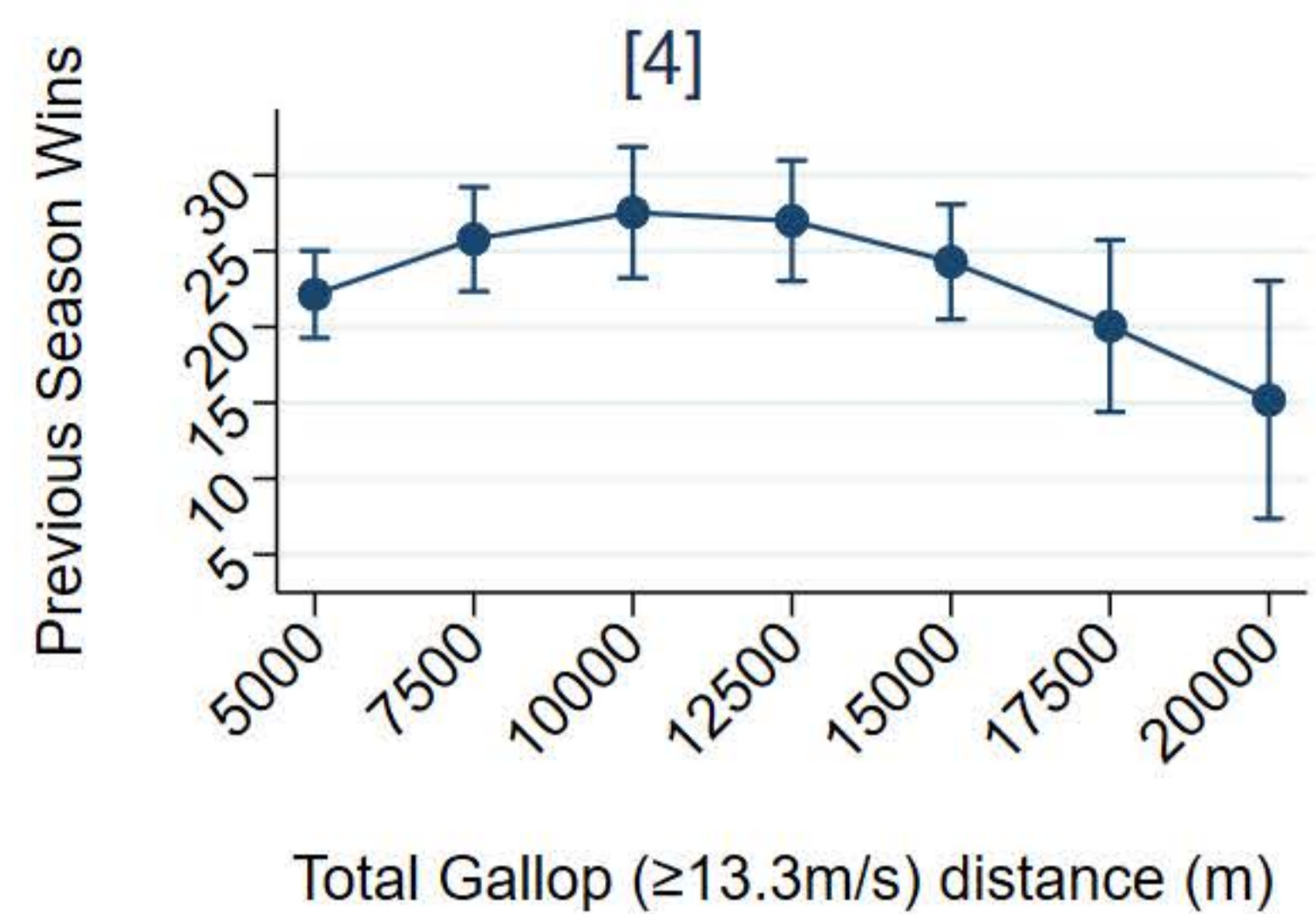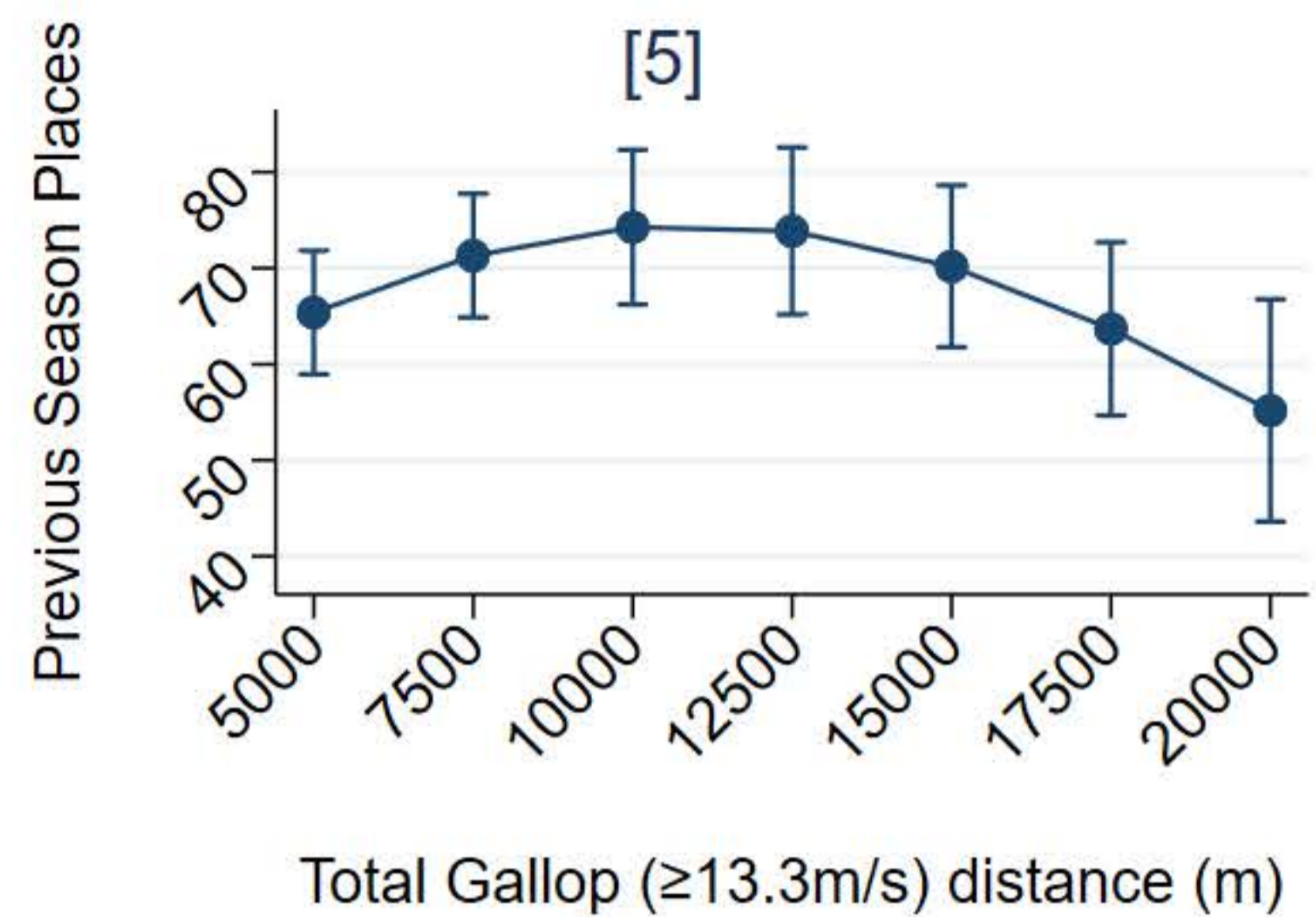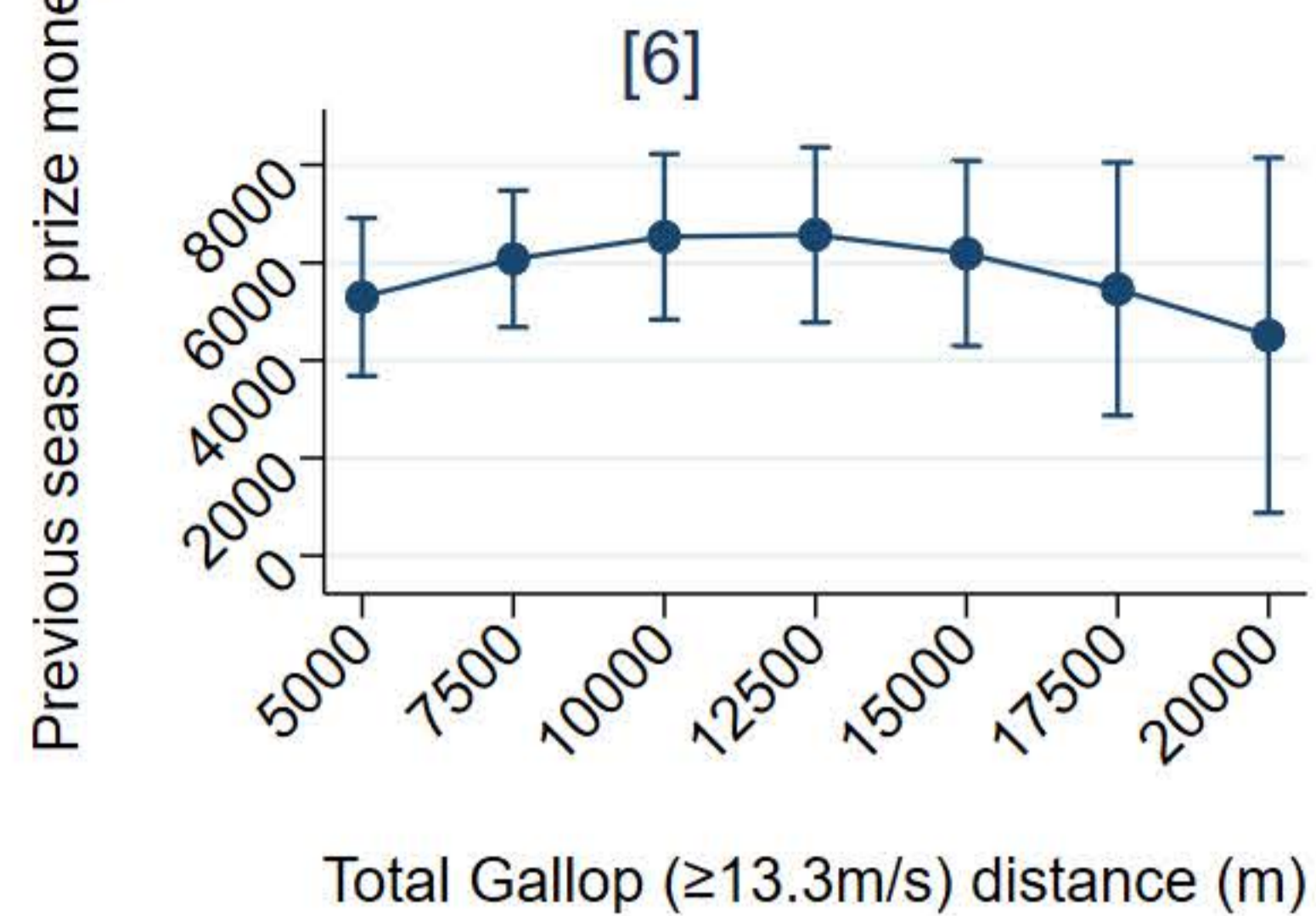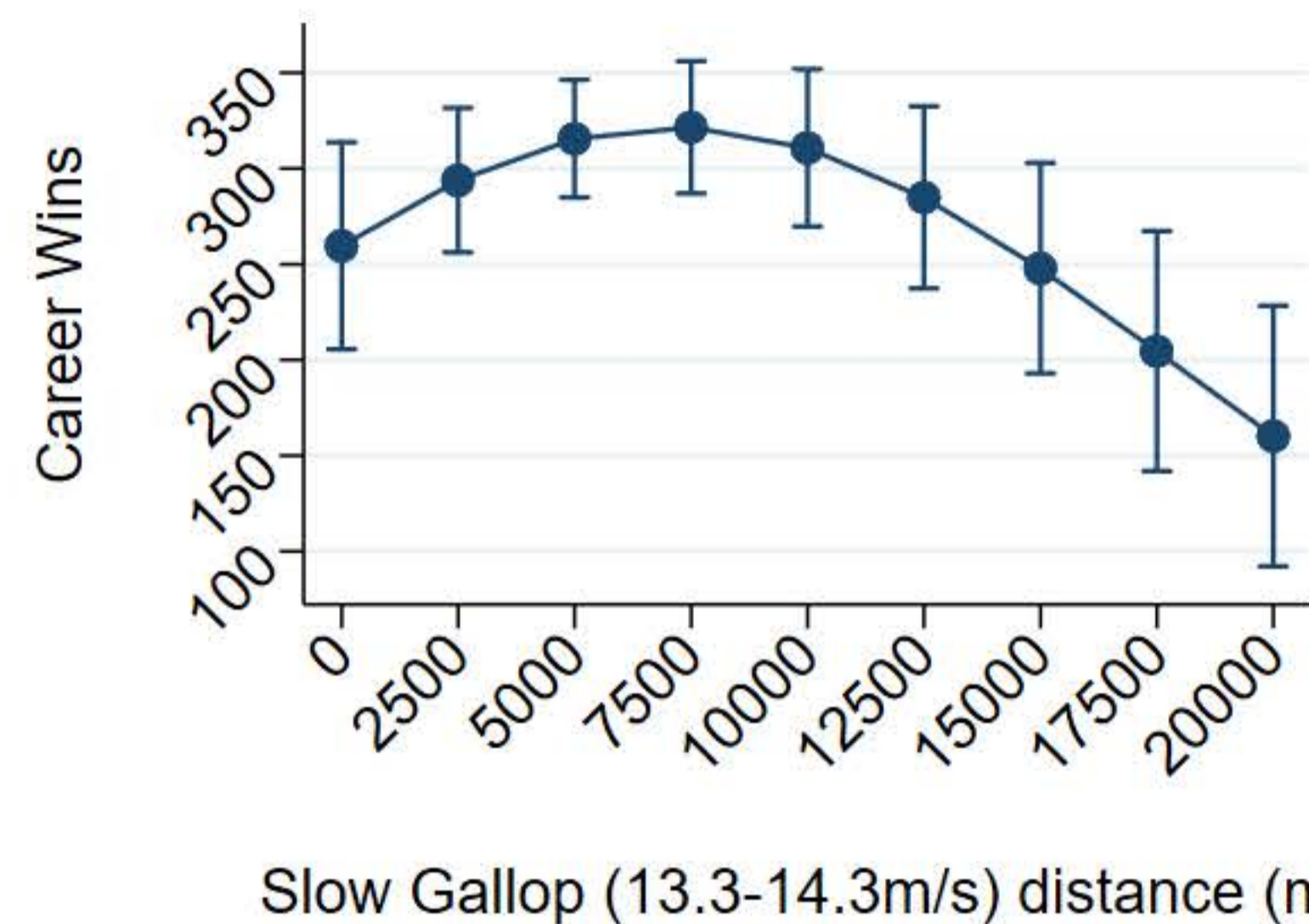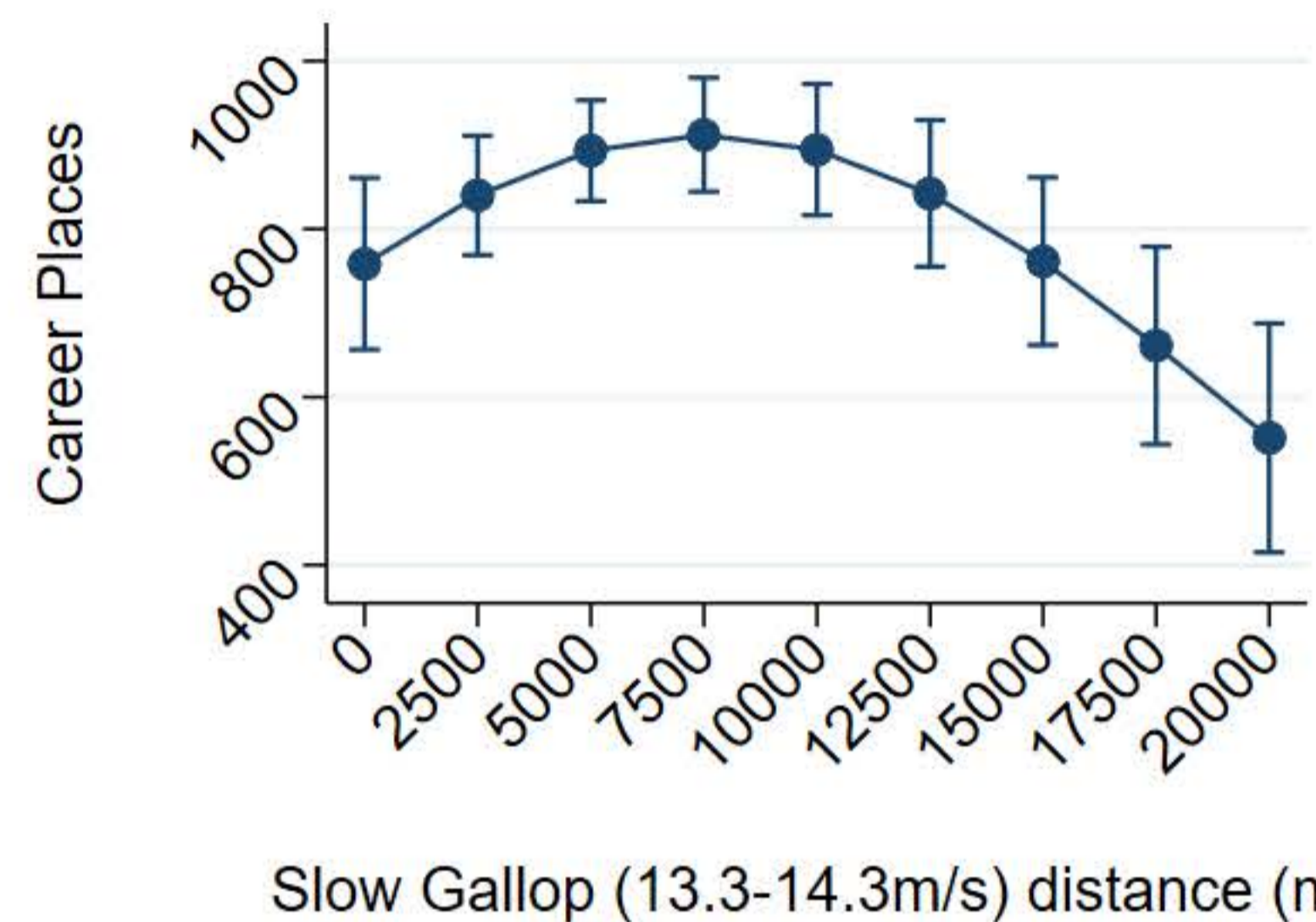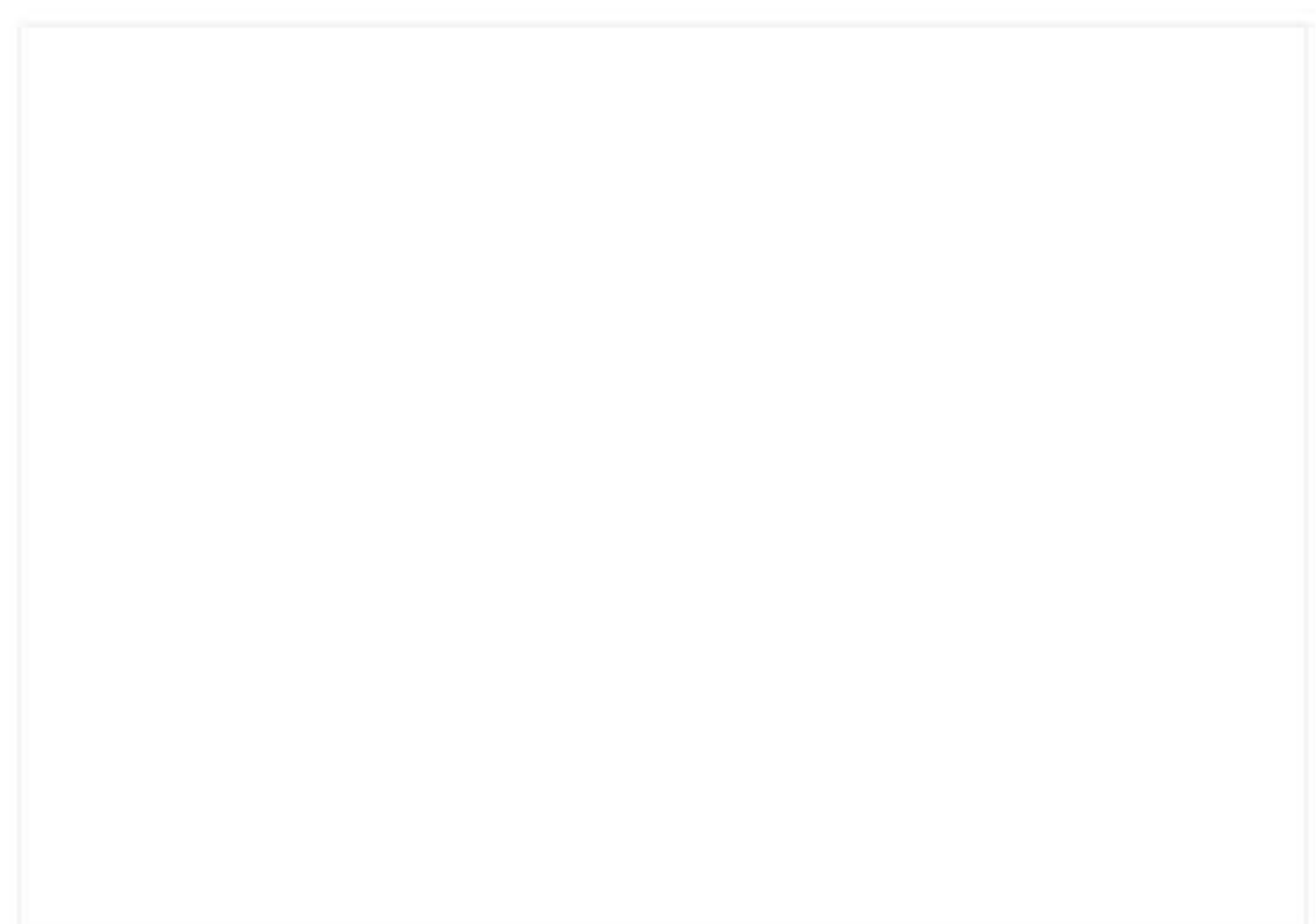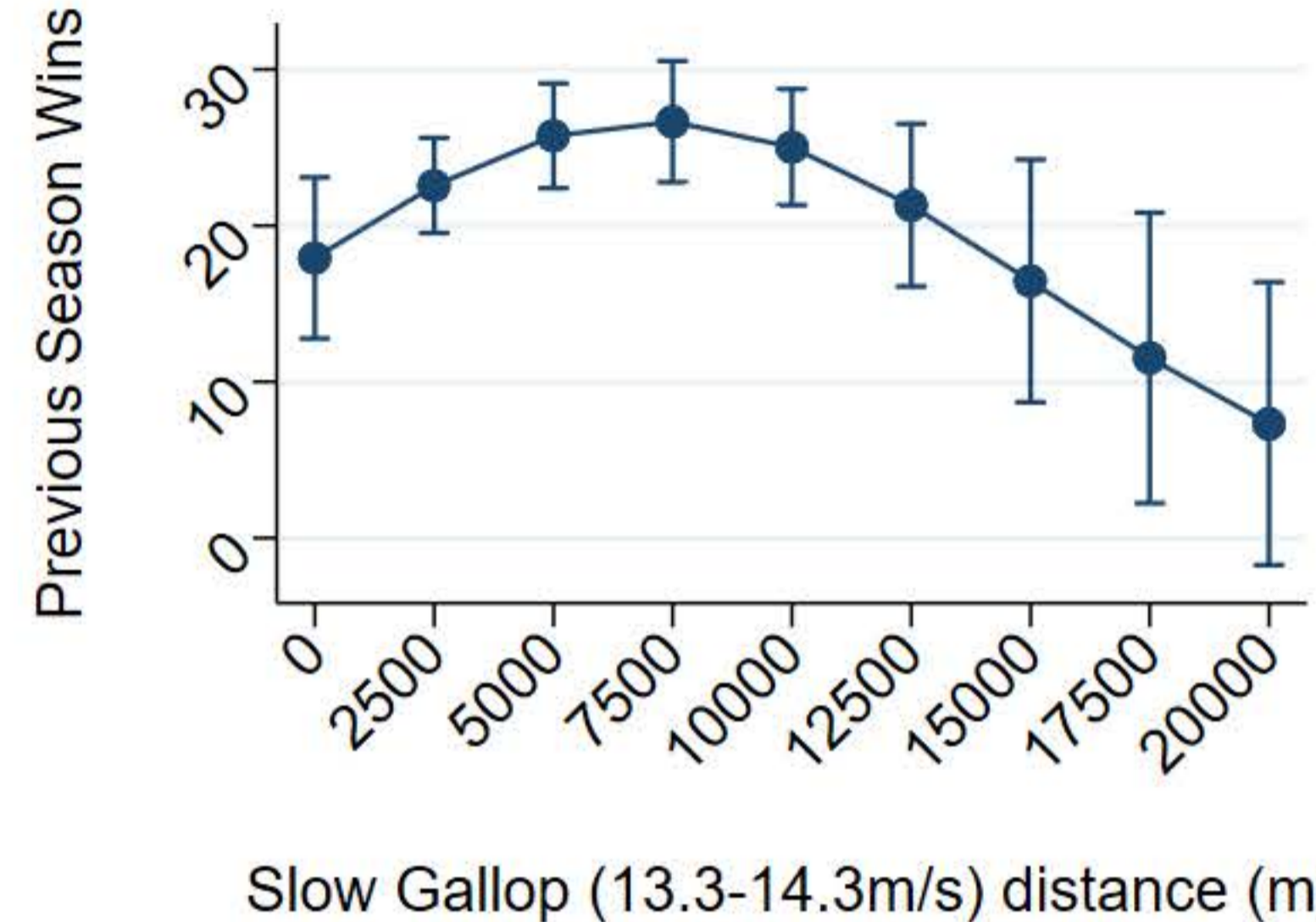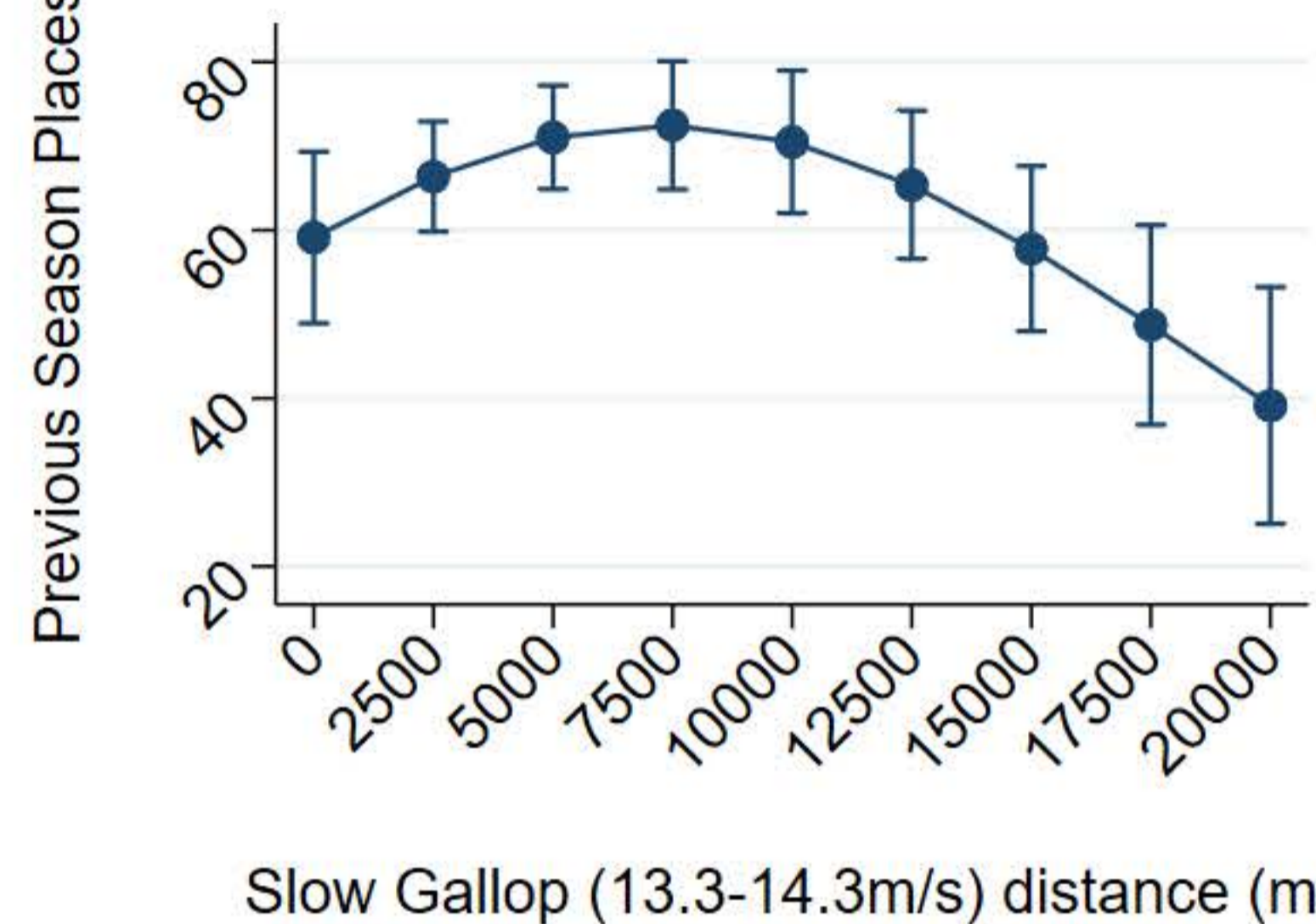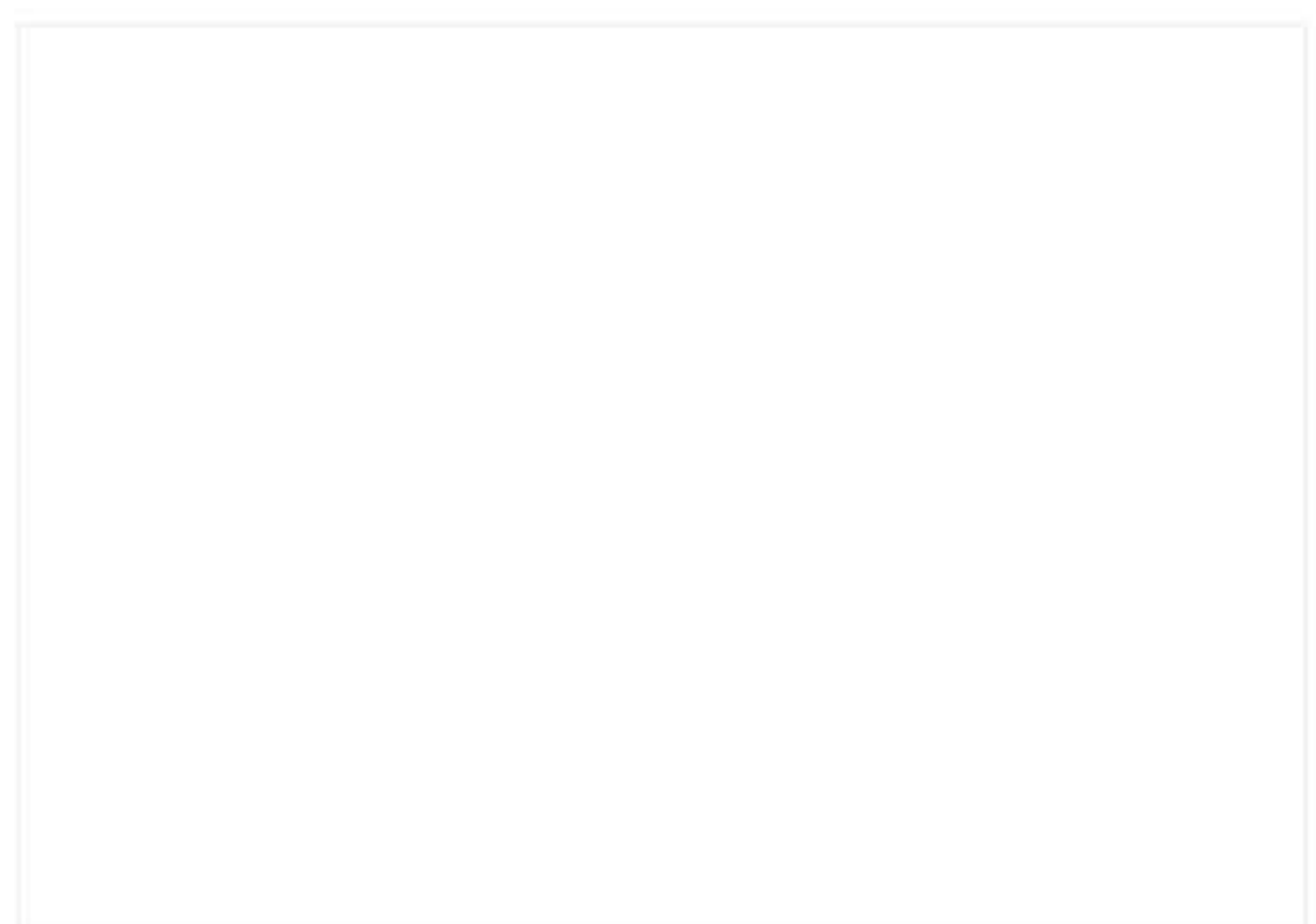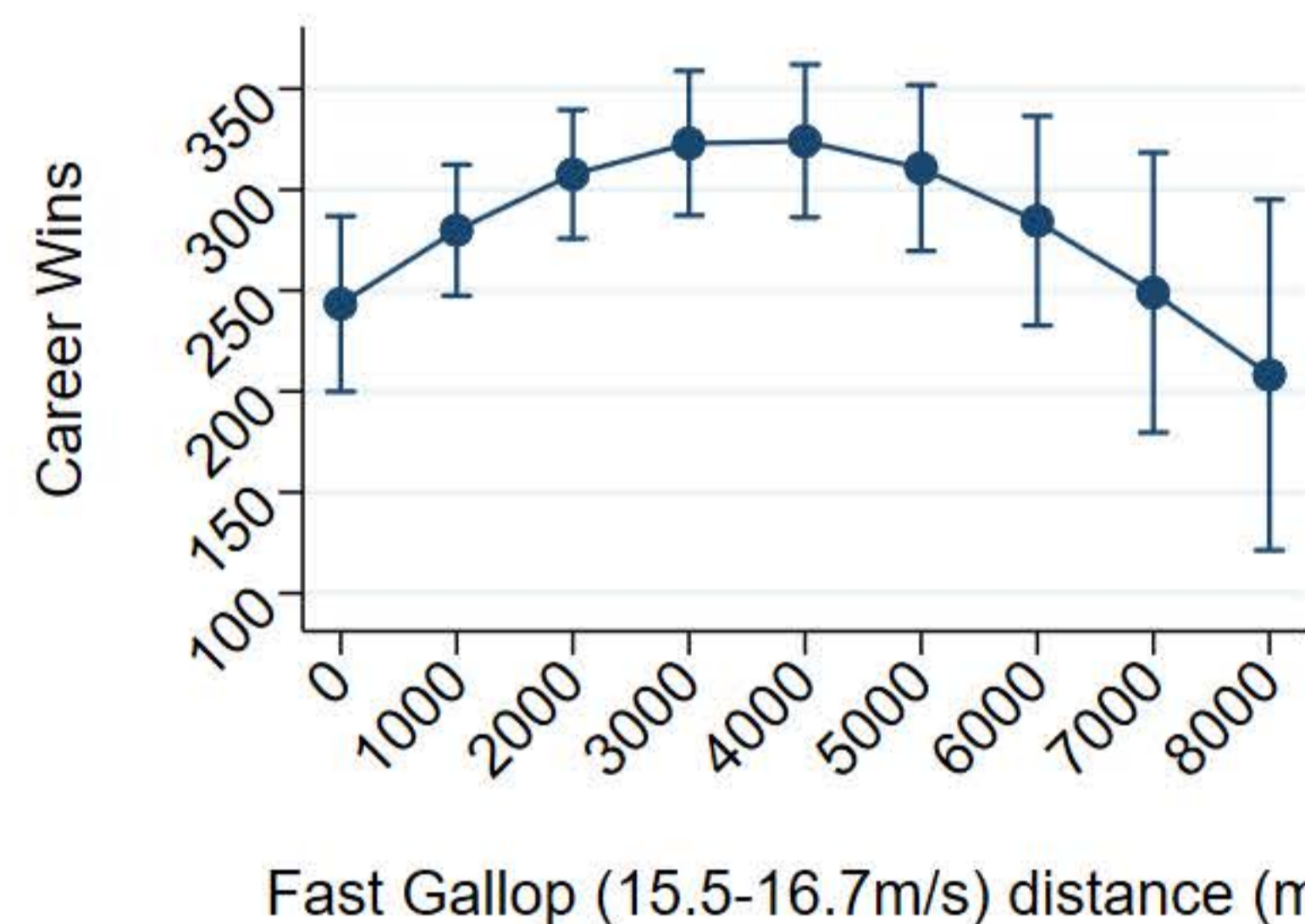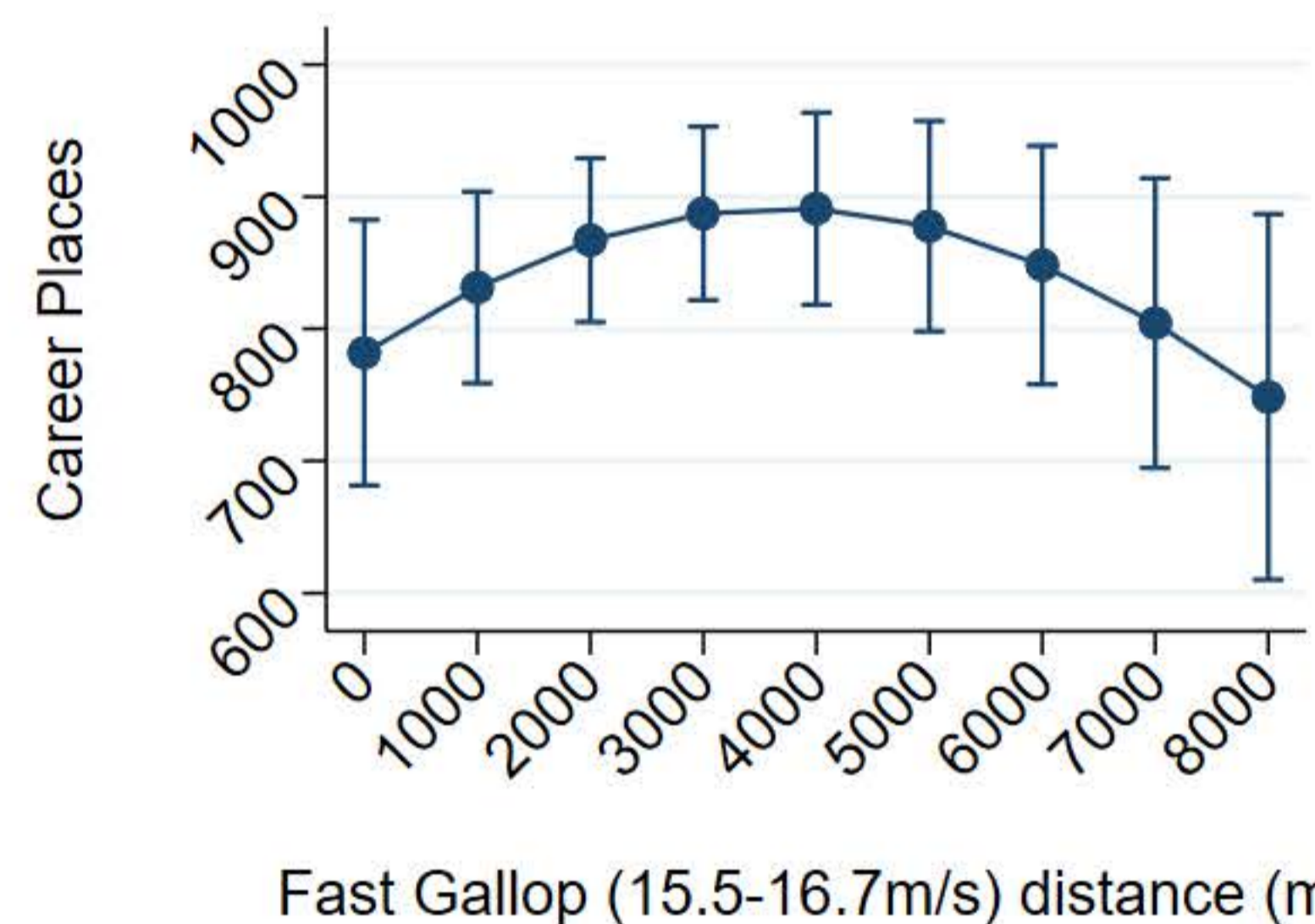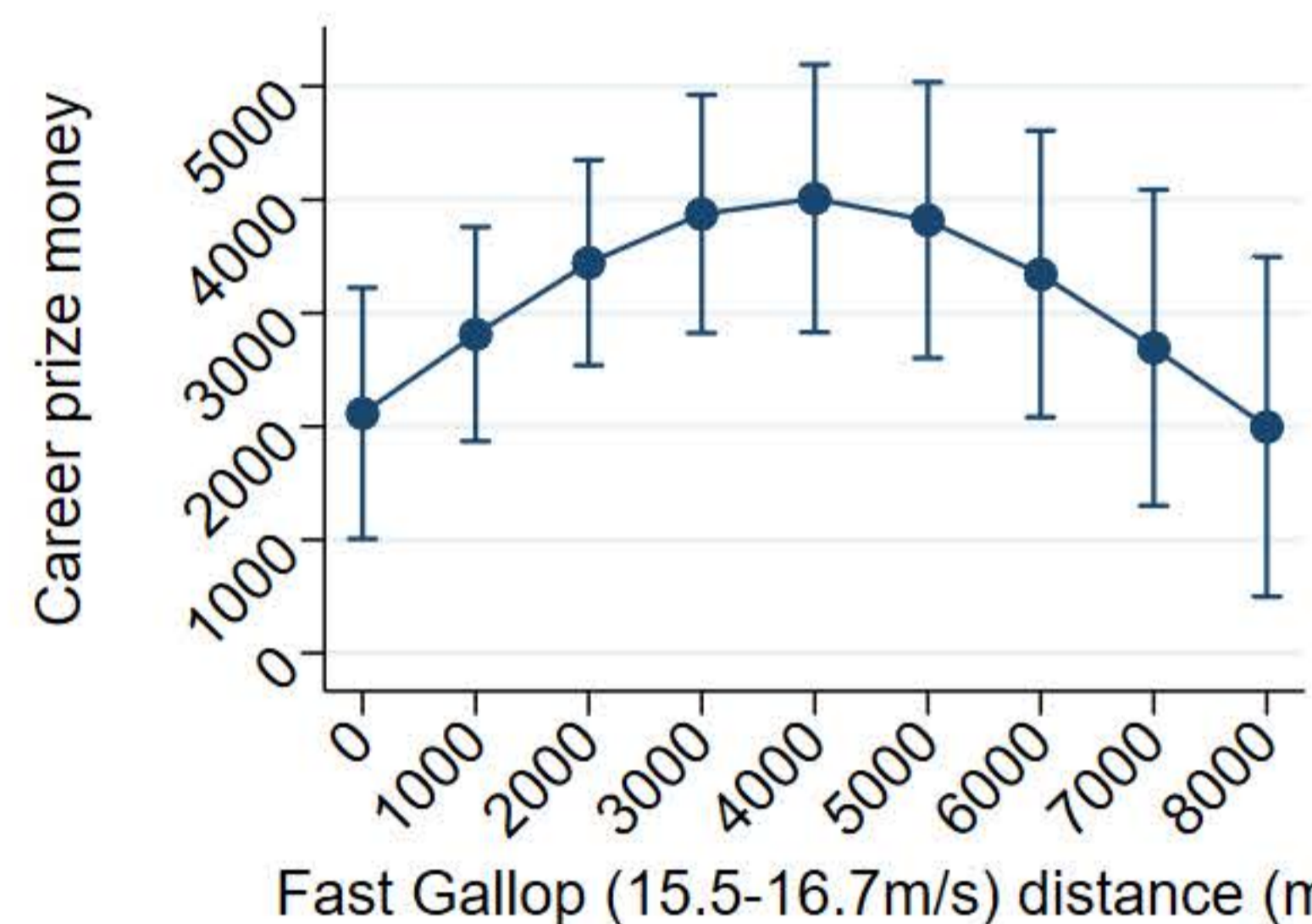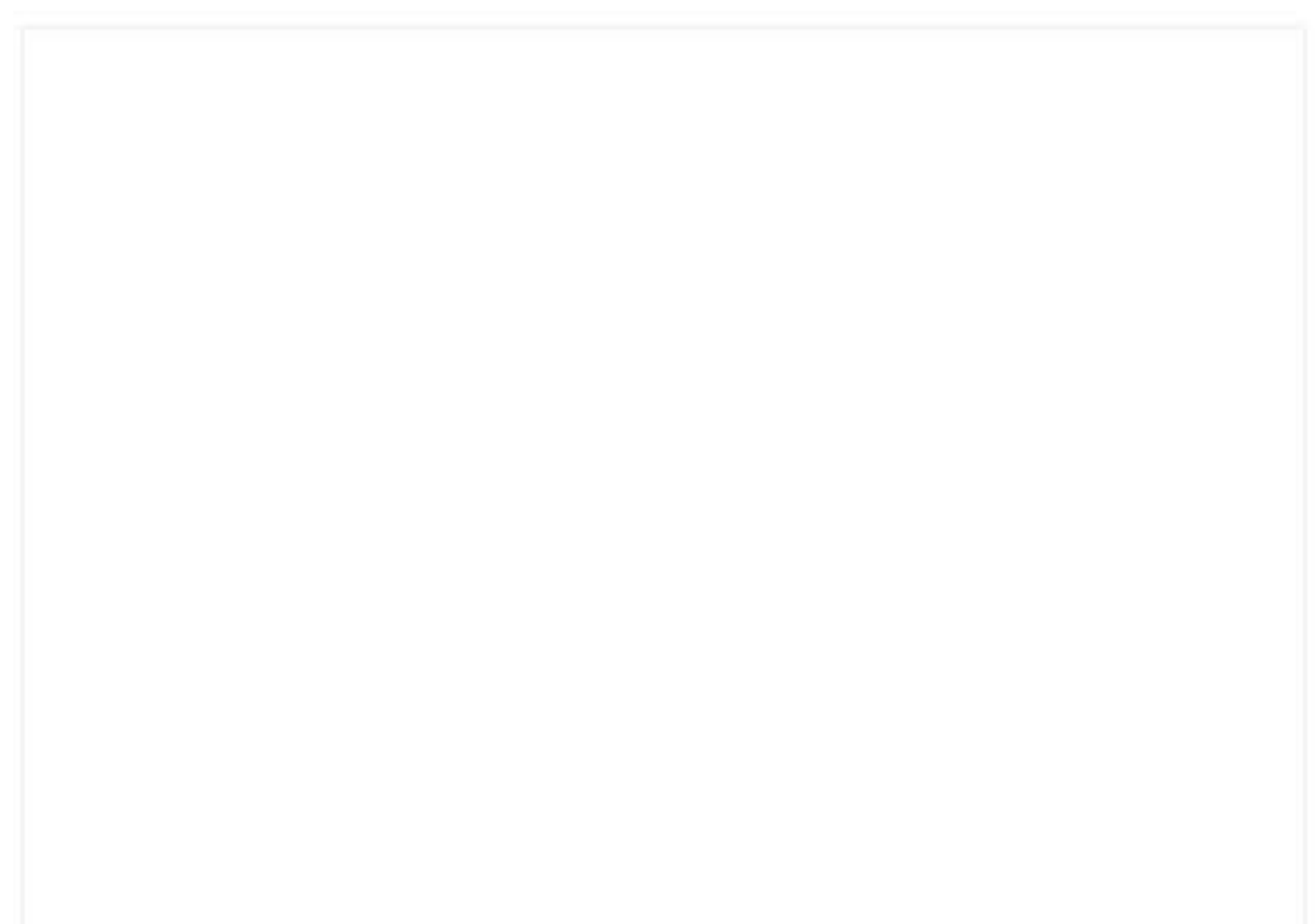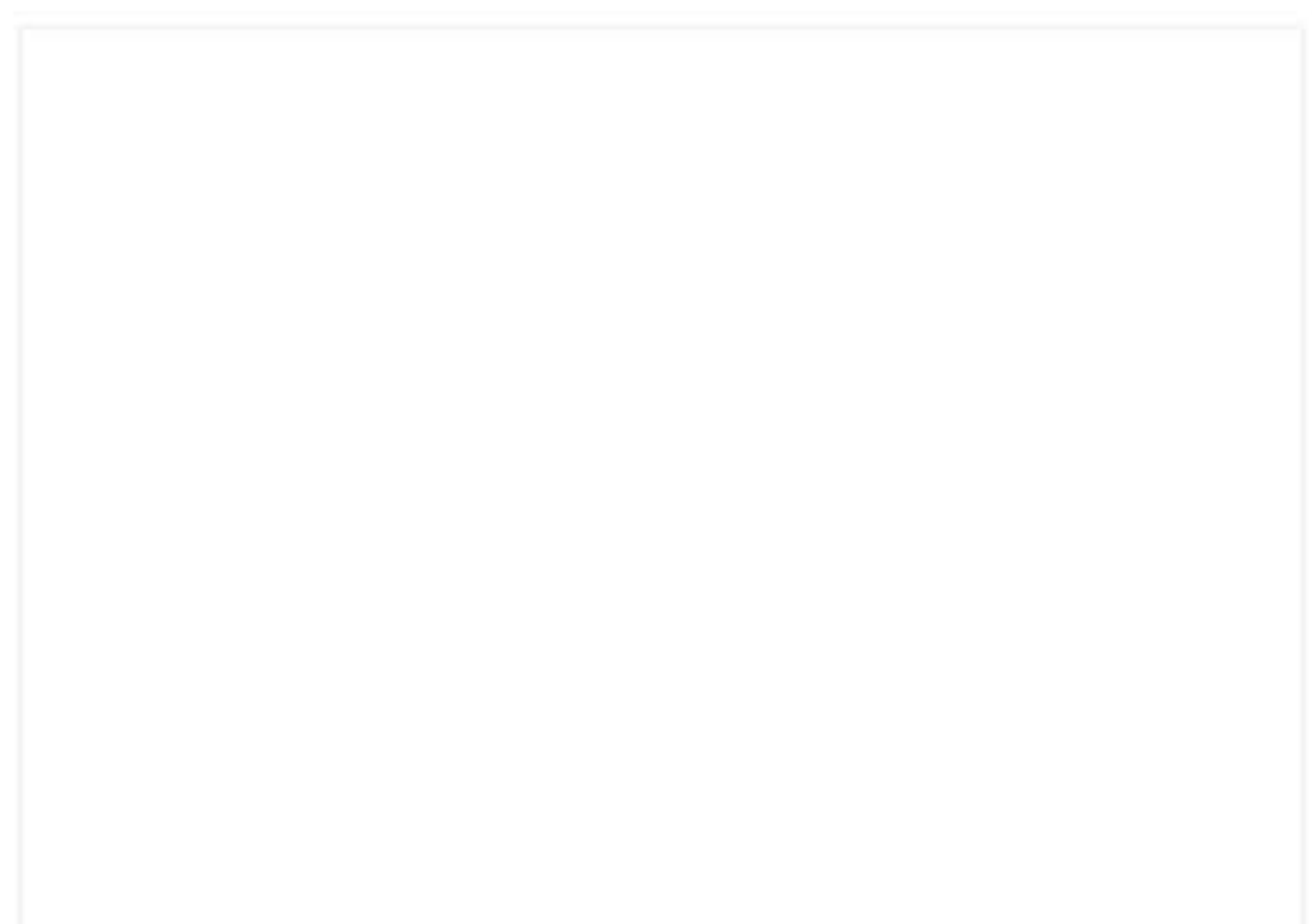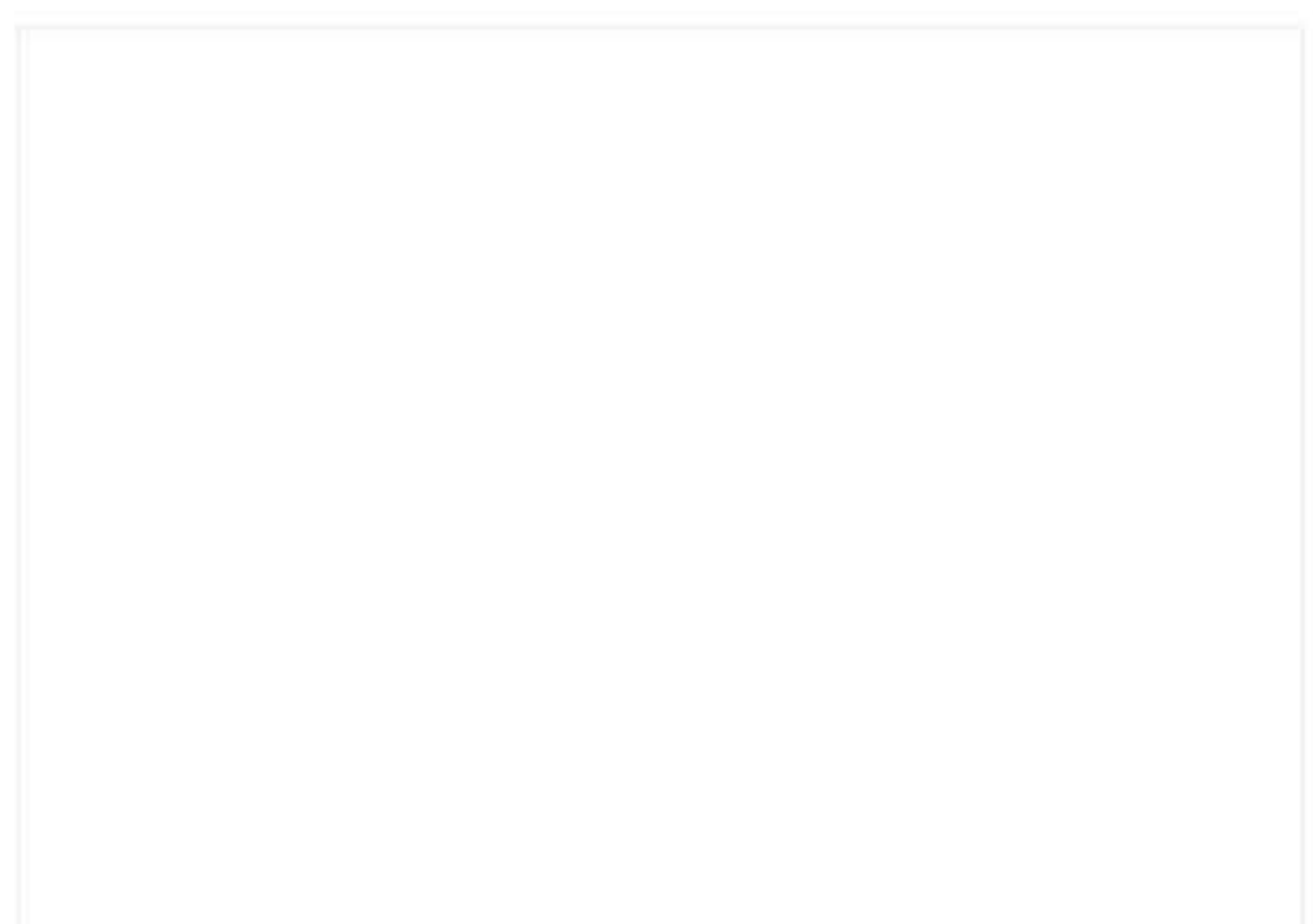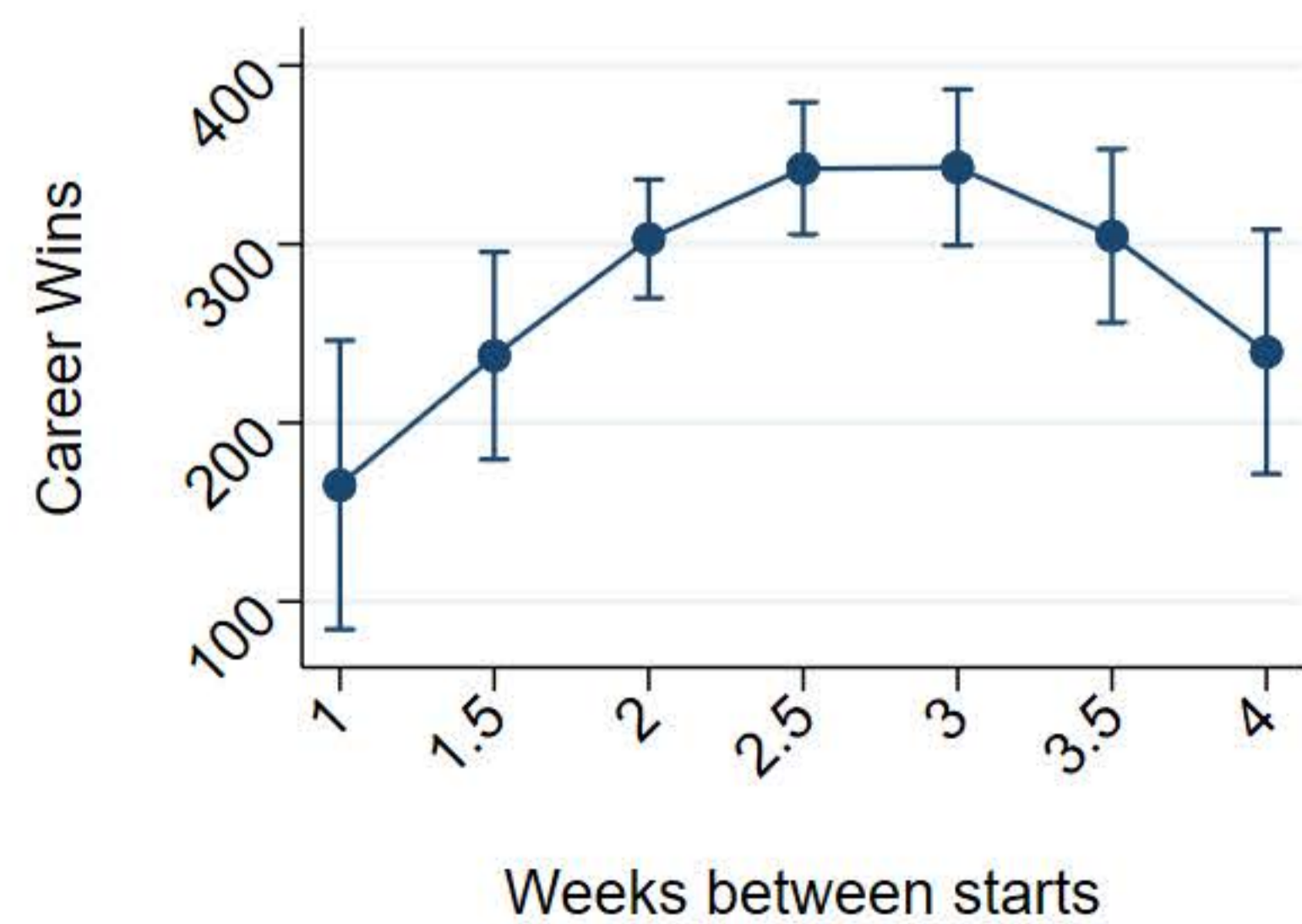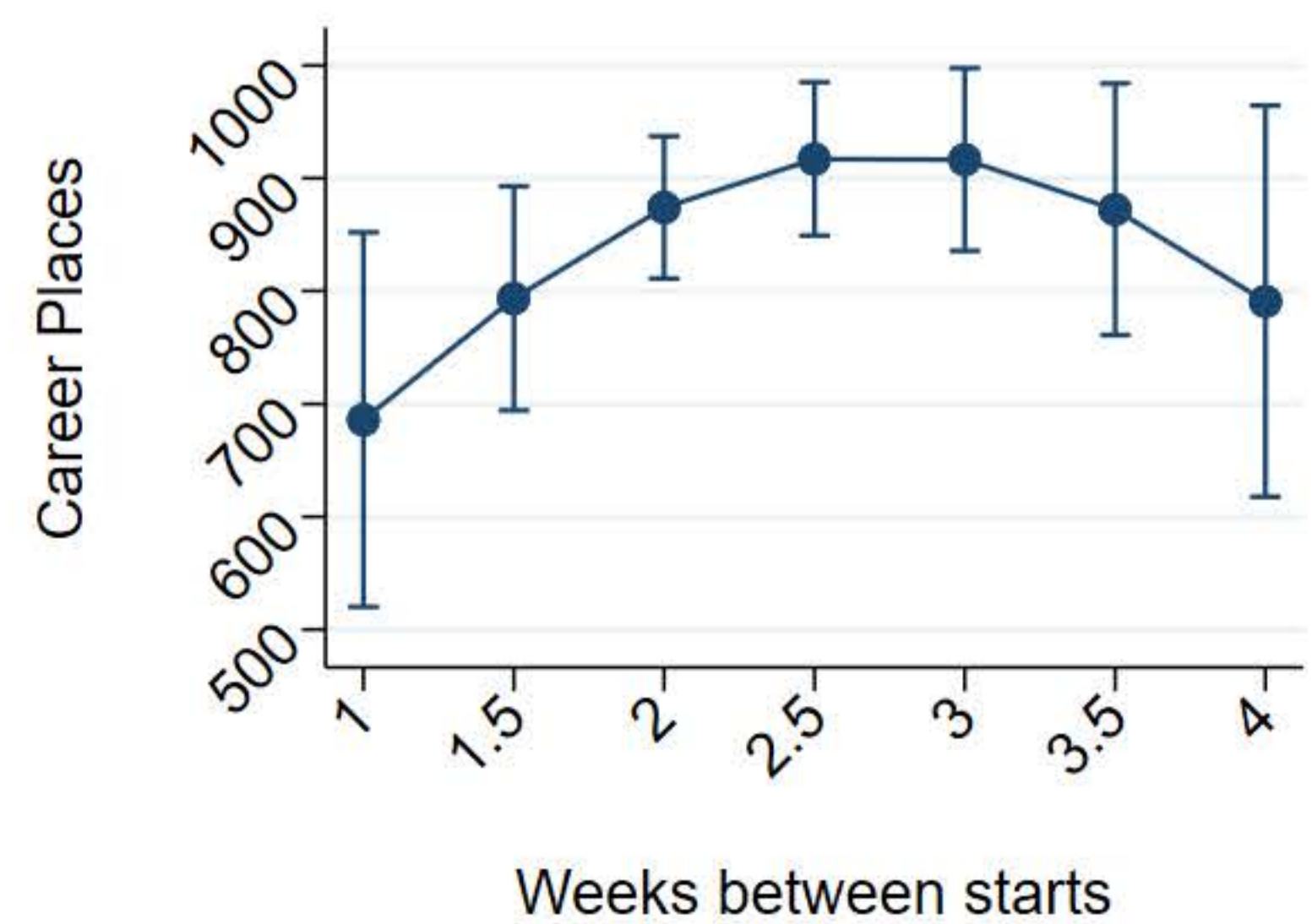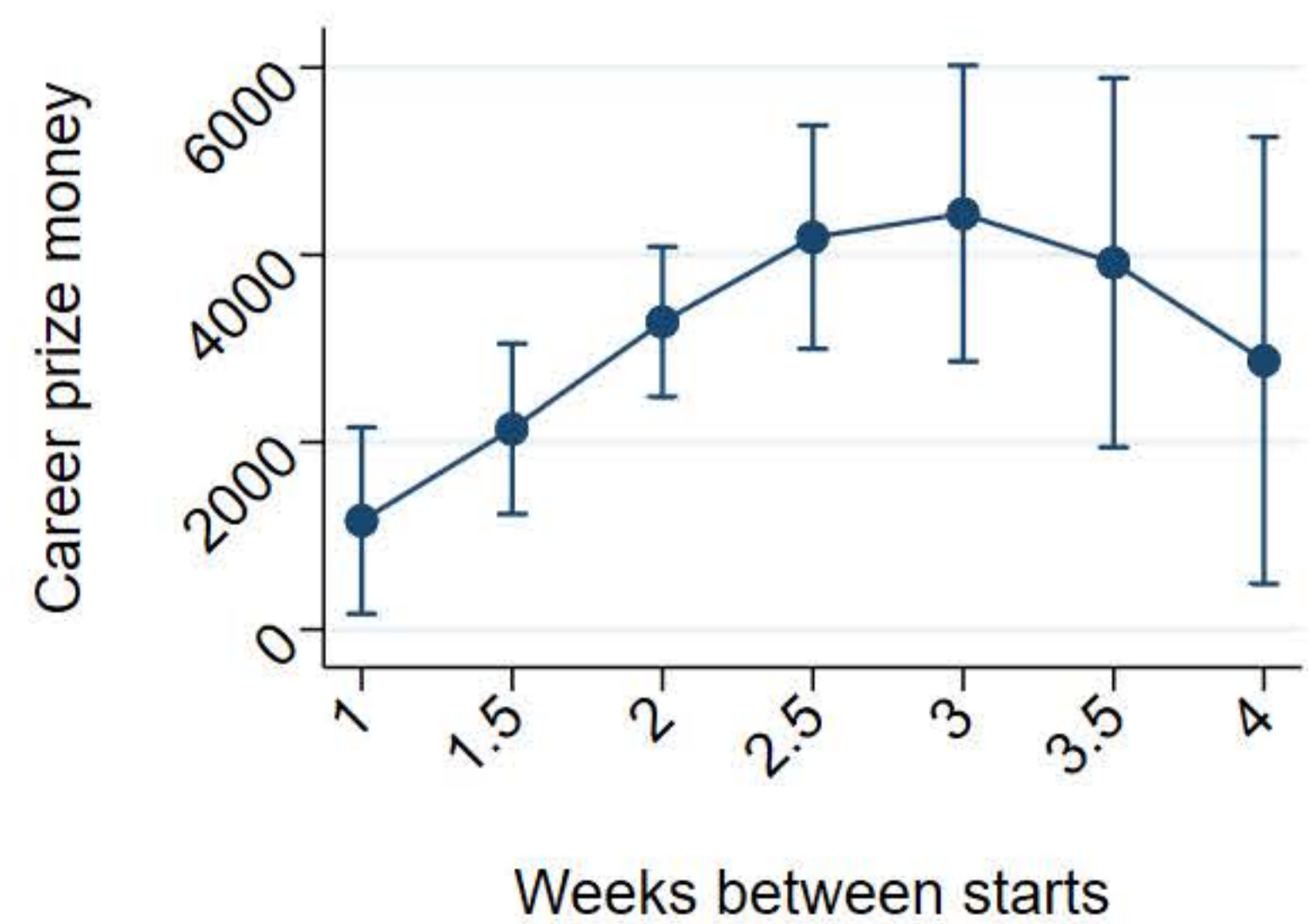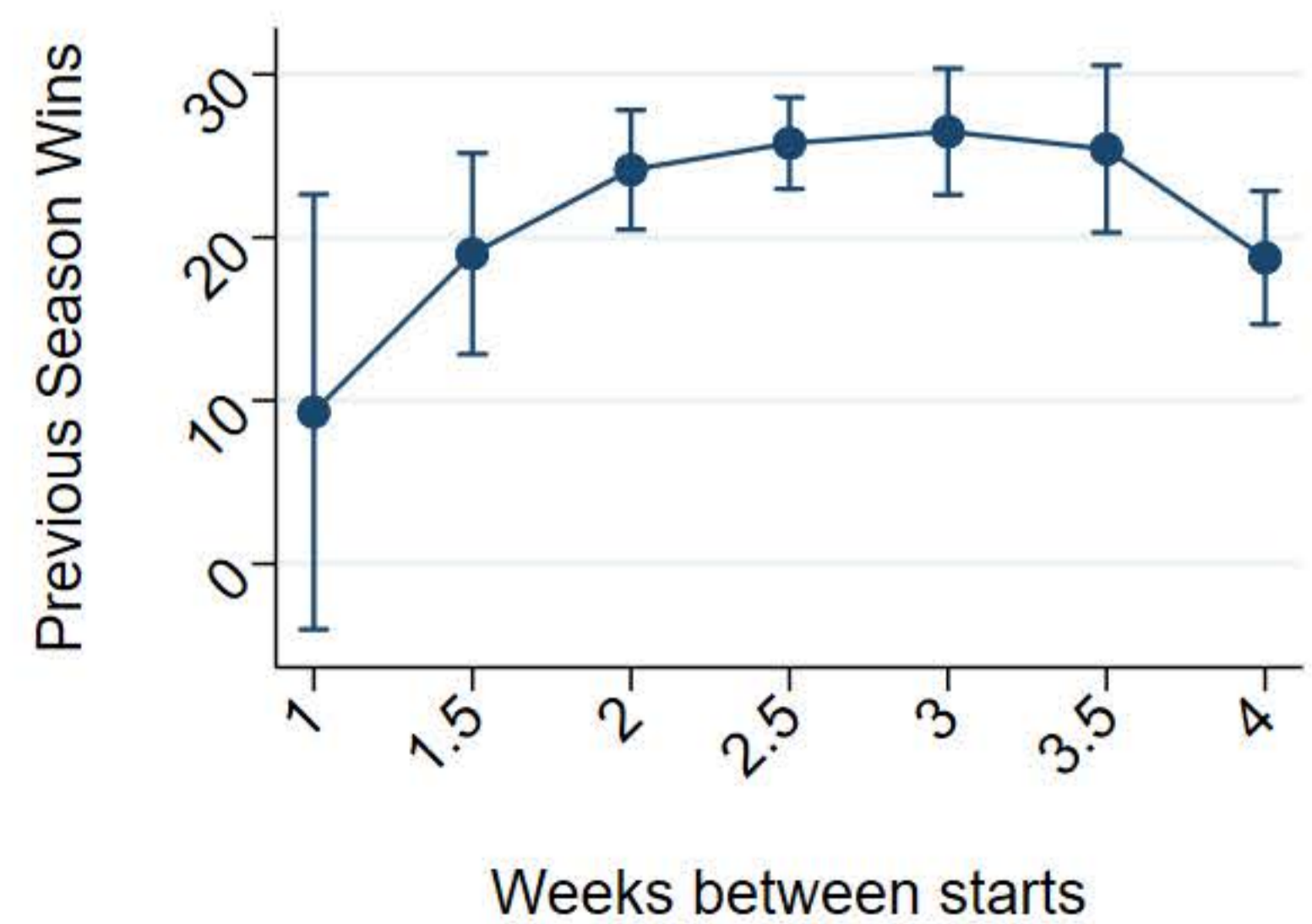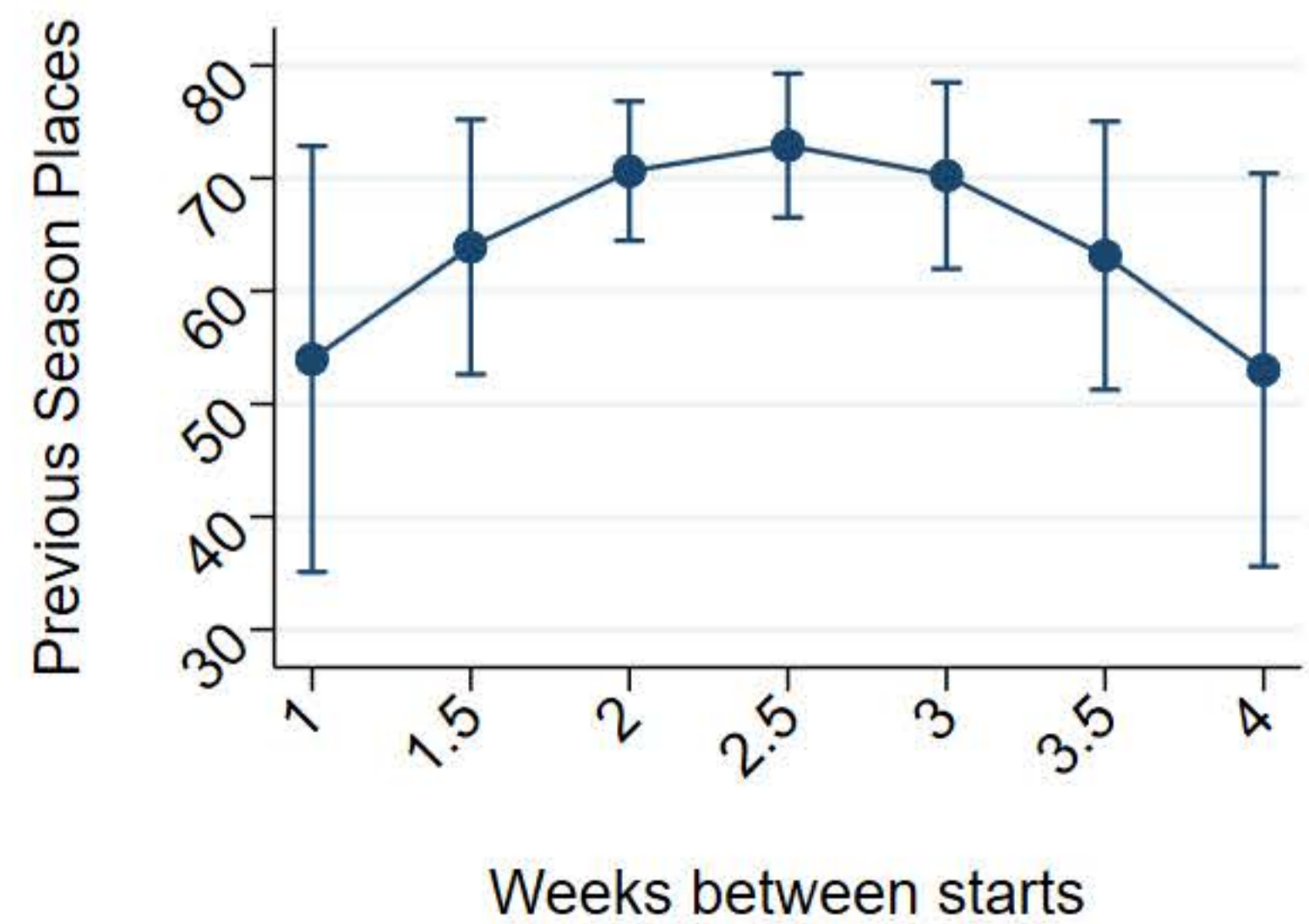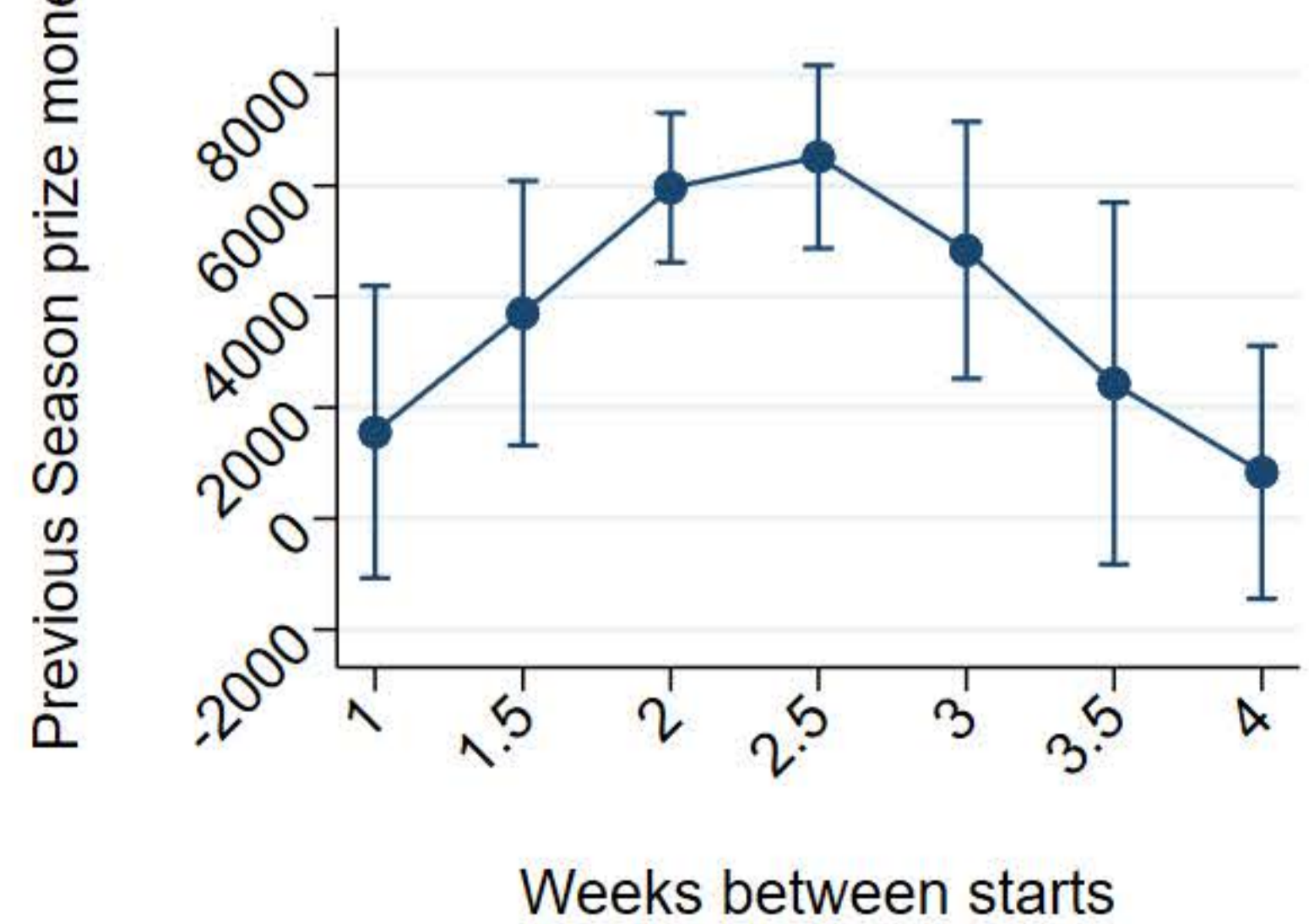

Supplement: Supplementary file 1 [file animals-11-03130-s001.zip › Supplementary figure S1.pdf]
